# Inhibition of TGFβ improves hematopoietic stem cell niche and ameliorates cancer-related anemia

**DOI:** 10.1186/s13287-020-02120-9

**Published:** 2021-01-18

**Authors:** Boyan Wang, Yi Wang, Hainan Chen, Senyu Yao, Xiaofan Lai, Yuan Qiu, Jianye Cai, Yinong Huang, Xiaoyue Wei, Yuanjun Guan, Tao Wang, Jiancheng Wang, Andy Peng Xiang

**Affiliations:** 1 Scientific Research Center, The Seventh Affiliated Hospital of Sun Yat-Sen University, 628# Zhenyuan Road, Shenzhen, Guangdong China; 2 Center for Stem Cell Biology and Tissue Engineering, Key Laboratory for Stem Cells and Tissue Engineering, Ministry of Education, Sun Yat-Sen University, 74# Zhongshan 2nd Road, Guangzhou, Guangdong China; 3 Department of Anesthesiology, The First Affiliated Hospital of Sun Yat-Sen University, Guangzhou, China; 4 Department of Hepatic Surgery and Liver Transplantation Center of the Third Affiliated Hospital, Organ Transplantation Institute, Sun Yat-Sen University, Guangzhou, China; 5 Department of Neurology, The Third Affiliated Hospital of Sun Yat-Sen University, Guangzhou, China; 6 Core Facility of Center, Zhongshan School of Medicine, Sun Yat-Sen University, Guangzhou, China; 7 Department of Biochemistry, Zhongshan School of Medicine, Sun Yat-Sen University, Guangzhou, China

**Keywords:** Cancer-related anemia, Cachexia, Erythropoiesis, Hematopoietic stem cells niche, Mesenchymal stromal cells, TGFβ, SB505124

## Abstract

**Background:**

Cancer cachexia is a wasting syndrome that is quite common in terminal-stage cancer patients. Cancer-related anemia is one of the main features of cancer cachexia and mostly results in a poor prognosis. The disadvantages of the current therapies are obvious, but few new treatments have been developed because the pathological mechanism remains unclear.

**Methods:**

C57BL/6 mice were subcutaneously injected with Lewis lung carcinoma cells to generate a cancer-related anemia model. The treated group received daily intraperitoneal injections of SB505124. Blood parameters were determined with a routine blood counting analyzer. Erythroid cells and hematopoietic stem/progenitor cells were analyzed by flow cytometry. The microarchitecture changes of the femurs were determined by micro-computed tomography scans. Smad2/3 phosphorylation was analyzed by immunofluorescence and Western blotting. The changes in the hematopoietic stem cell niche were revealed by qPCR analysis of both fibrosis-related genes and hematopoietic genes, fibroblastic colony-forming unit assays, and lineage differentiation of mesenchymal stromal cells.

**Results:**

The mouse model exhibited hematopoietic suppression, marked by a decrease of erythrocytes in the peripheral blood, as well as an increase of immature erythroblasts and reduced differentiation of multipotent progenitors in the bone marrow. The ratio of bone volume/total volume, trabecular number, and cortical wall thickness all appeared to decrease, and the increased osteoclast number has led to the release of latent TGFβ and TGFβ signaling over-activation. Excessive TGFβ deteriorated the hematopoietic stem cell niche, inducing fibrosis of the bone marrow as well as the transition of mesenchymal stromal cells. Treatment with SB505124, a small-molecule inhibitor of TGFβ signaling, significantly attenuated the symptoms of cancer-related anemia in this model, as evidenced by the increase of erythrocytes in the peripheral blood and the normalized proportion of erythroblast cell clusters. Meanwhile, hindered hematopoiesis and deteriorated hematopoietic stem cell niche were also shown to be restored with SB505124 treatment.

**Conclusion:**

This study investigated the role of TGFβ released by bone remodeling in the progression of cancer-related anemia and revealed a potential therapeutic approach for relieving defects in hematopoiesis.

## Background

Cancer cachexia is an irreversible but common wasting syndrome in terminal stage cancer patients that is characterized by weight loss, anorexia, asthenia, and anemia [1–3]. Although a portion of cancer-related anemia (CRA) is secondary to antineoplastic treatment, primary CRA is developed in more than 30% of patients [4, 5]. Patients with CRA exhibit fatigue, lethargy, dyspnea, anorexia, and progressive worsening of cognitive function, which adversely influence their quality of life and the range of sustainable treatments, ultimately decreasing the survival of these patients [6, 7]. However, the etiology of CRA has not yet been elucidated, and the mechanisms underlying its progression are unclear, which complicates the diagnosis and treatment of CRA.

Red blood cells (RBC) are generated from hematopoietic stem cells (HSC) through a stepwise differentiation process called erythropoiesis. The earliest committed erythroid progenitor cell is the erythroid burst-forming units (BFU-E) [8]. BFU-E is largely dormant but capable of differentiating into erythroid colony-forming units (CFU-E) [9]. CFU-E gives rise to proerythroblasts (pro-E), with an absolute requirement for erythropoietin (EPO). Pro-E undergoes successive maturation stages, including basophilic, polychromatic, and orthochromatic erythroblasts, before finally becoming reticulocytes and then RBC [8]. In recent years, the work of many researchers has revealed that hematopoiesis is a complex process that is strictly regulated by the surrounding endosteal and stromal niches [10, 11]. The endosteal niche, which mainly comprises osteoblasts and osteoclasts, usually exists in a dynamic balance between bone formation and resorption, which is called bone remodeling. Bone remodeling activity is closely associated with hematopoiesis, which can regulate the proliferation, differentiation, and long-term erythropoietic capacity of HSC both directly and indirectly [12]. Additionally, the stromal niche forms parts of the bone marrow microenvironment and influences the steady-state and stress-induced proliferation and differentiation of erythroid progenitor cells [13–15].

In the hematopoietic compartment, the transforming growth factor β (TGFβ) signaling pathway is an important regulator of proliferation and differentiation of different cell types, and it has been implicated in the pathogenesis of a wide variety of bone marrow disorders [16]. Bone matrix is the major source of TGFβ in the bone marrow [17]. During the deposition of the bone matrix, osteoblasts produce TGFβ in a latent form that binds with the bone matrix [18]. During bone resorption, this latent TGFβ is released from the bone matrix and then cleaved by osteoclasts to become active TGFβ [19]. TGFβ is a double-edged sword in this process: activated TGFβ can promote the migration of bone marrow stromal cells into the bone resorptive sites and induce bone formation [19, 20], but excess TGFβ from the bone can be a pathological mechanism for multiple diseases [21]. Some researchers have demonstrated that the muscle weakness observed in cancer patients is related to the osteolytic processes of some invasive tumors that release large amounts of TGFβ during bone destruction [22]. In patients with Camurati-Engelmann disease, for example, TGFβ is secreted by osteoblasts and then activated without binding to the bone matrix, leading to severe hyperostosis and osteoarthritis due to abnormal osteogenesis [23, 24]. However, the potential role of TGFβ in the pathogenesis of CRA has not yet been explored.

In this study, we confirmed that Lewis lung carcinoma (LLC)-bearing mice showed a reduction of erythrocytes and hemoglobin in the peripheral blood and suppression of hematopoiesis in the bone marrow. In addition, we observed increased bone resorption, activated TGFβ signaling, and deteriorated HSC niches. Furthermore, blockage of the TGFβ signaling by SB505124 attenuated the deterioration of HSC niche and hematopoiesis and subsequently improved the symptoms of CRA in both the peripheral blood and bone marrow. Our results show that the TGFβ pathway plays an important role in the development of CRA and suggest that TGFβ signaling inhibition could be an attractive strategy for treating this condition.

## Methods

### Animals

C57BL/6 mice were obtained from the Guangdong Medical Laboratory Animal Center (Guangzhou, China). Lewis lung carcinoma (LLC) cell line (Chinese Academy of Sciences) were counted and resuspended in sterilized phosphate-buffered saline (PBS). Homozygous transgenic mice expressing enhanced GFP controlled by a Nestin promoter (Nestin-GFP, on the C57BL/6 genetic background) were kindly provided by Dr. Masahiro Yamaguchi [25]. 2 × 10^6^ LLC cells were suspended in 100 μl sterilized PBS, or the vehicle was subcutaneously injected into the left flanks of 8-week-old male mice. All animals received the intraperitoneal injections of either 100 μl vehicle (100% DMSO) [26, 27] or SB505124-dissolved (5 mg/day/kg, Selleck) DMSO (Sigma) from day 7 to day 21 since tumor implantation [23]. All animal procedures were performed in accordance with the animal care guidelines of the National Institutes of Health (NIH) and under protocols approved by the Ethical Committee of Sun Yat-Sen University.

### Cell culture experiments

LLC cells were purchased from the Chinese Academy of Sciences (Shanghai, China) and cultured in Dulbecco’s modified Eagle’s medium (DMEM) (high glucose, Gibco) supplemented with 10% fetal bovine serum (FBS) (PAN-Biotech) and 1× penicillin/streptomycin (Invitrogen) at 37 °C and 5% CO_2_. All cell lines tested negative for mycoplasma contamination.

### Routine examination of the blood

Blood samples were extracted from the inferior vena cava of mice under anesthesia, with 5 mM EDTA (pH = 8.0) as an anticoagulant. Each sample was immediately sent to the Third Affiliated Hospital, Sun Yat-Sen University, and tested routine blood parameters by a routine blood counting analyzer (Beckman Coulter, Fullerton).

### RNA isolation and quantitative PCR

Gene expression was assessed by qPCR as previously described [28]. Briefly, total RNA was extracted from cell lysates using the TRIzol reagent (Molecular Research Center, Inc.). First-strand cDNA was synthesized with a RevertAid First Strand cDNA Synthesis Kit (Thermo) according to the manufacturer’s instructions, and qPCR was performed with the LightCycler 480 SYBR Green I Master Mix (Roche) and a Light Cycler 480 Detection System (Roche). The level of each target mRNA was normalized with respect to that of the 18s rRNA. The sequences of the primers used for qPCR are listed in Supplemental Table S1.

### Western blotting

For Western blotting, cells extracted from the bone marrow were stained with antibodies against Ter119 (eBioscience), and Ter119^+^ cells were sorted and collected with a BD Influx flow cytometer. The collected cells were washed twice with cold PBS, directly lysed in 1× RIPA buffer (Millipore) supplemented with protease inhibitor cocktail (Roche) and phosphatase inhibitor cocktail (Roche), and then centrifuged at 15,000*g* for 5 min at 4 °C. Each supernatant was recovered as a total cell lysate. Equal amounts of protein were resolved by SDS-PAGE and then electrotransferred to a 0.45-μm pore-sized polyvinylidene difluoride (PVDF) membrane (Millipore). Specifically bound primary antibodies were detected using horseradish peroxidase (HRP)-coupled secondary antibodies and enhanced chemiluminescence (Millipore). The utilized primary and secondary antibodies are listed in Supplemental Table S2.

### Cell sorting and flow cytometry

Erythroid differentiation was monitored using monoclonal antibodies against CD44 (eBioscience) and Ter119 (eBioscience) by flow cytometry, as described previously [9]. Briefly, we collected the bone marrow from the femoral cavities of the mice by flushing it with a 25-gauge needle. Cells extracted from the bone marrow were suspended in PBS containing 1% BSA and 1 mM ethylenediaminetetraacetic acid (EDTA, pH = 8.0), pretreated with CD16/32 antibodies (eBioscience) for 30 min at 4 °C, and then incubated with antibodies against CD44 (eBioscience) and Ter119 (eBioscience) for 30 min at 4 °C in the dark. DAPI (Roche) counterstaining was performed right before the analysis, and dead cells were excluded. To analyze the long-term/short-term hematopoietic stem cells (LT/ST HSC), multipotent progenitors (MPP), common myeloid progenitors (CMP), and common lymphoid progenitors (CLPs), we extracted the bone marrow cells as mentioned above. Lysis of red blood cells was performed with RBC Lysis Buffer (#64010-00-100, Biogems) under the manufacturer’s procedure. Cells were stained with Lineage Antibody Cocktail (eBioscience) and antibody against Sca-1 (eBioscience), CD117 (c-kit) (eBioscience), CD34 (eBioscience), CD16/32 (eBioscience), CD127 (eBioscience), and CD135 (eBioscience) for 30 min at 4 °C in the dark. For the analysis of myeloid cell proportion, bone marrow cells were extracted and processed with RBC Lysis Buffer (#64010-00-100, Biogems) without immunostaining [29, 30]. Cells were washed twice with PBS containing 1% BSA and 1 mM EDTA (pH = 8.0) and analyzed on a CytoFLEX flow cytometer (Beckman Coulter).

The bone marrow of Nestin-GFP mice was extracted as mentioned above. Lysis of red blood cells was performed with RBC Lysis Buffer (#64010-00-100, Biogems) under the manufacturer’s procedure. After that, cells were stained with Ter119 (eBioscience), CD45 (eBioscience), and CD31 (eBioscience) for 30 min at 4 °C in the dark. Cells were washed twice with PBS containing 1% BSA and 1 mM EDTA (pH = 8.0). Cell sorting was performed using Influx Cell Sorter (Becton Dickinson). CD31^−^ CD45^−^ Ter119^−^ Nestin-GFP^+^ cells were sorted.

The data were processed using the FlowJo (Tree Star) or CytExpert (Beckman Coulter) software packages. The utilized antibodies are listed in Supplemental Table S2.

### Immunostaining and confocal imaging of bone marrow femoral sections

The femoral sections were prepared, immunostained, and imaged as previously described [31]. Bones were fixed overnight in 4% paraformaldehyde and decalcified for 2 weeks in 10% EDTA (pH = 8.0). The longitudinal bone sections were stained overnight at 4 °C with primary antibodies against Runx2, p-Smad2/3 (Ser423/425), and smooth muscle actin and counterstained with DAPI (Roche). The primary antibodies were detected with goat anti-rabbit IgG Alexa 555 or donkey anti-goat IgG Alexa 594 as appropriate. Bone imaging was performed on an Andor Dragonfly CR-DFLY-202-40.

### Micro-computed tomography

In vitro high-resolution micro-computed tomography (micro-CT) images were obtained using an Inveon PET/CT scanner (Siemens). We dissected the femur from control or LLC-bearing mice and fixed them in 4% PFA for 48 h. The Inveon Research Workplace 4.1 software was used to reconstruct and analyze the images. The whole subchondral bone medial compartment was defined as the reconstruction area, and three-dimensional structure analysis was performed. The three-dimensional structural parameters analyzed included trabecular bone volume per tissue volume (BV/TV), bone surface area/TV (bone surface area per tissue volume), trabecular number (Tb.Nu), trabecular pattern factor (Tb.Pf), trabecular thickness (Tb.Th), trabecular separation (Tb.Sp), and cortical wall thickness.

### Trap staining and analysis

Paraffin sectioning and Trap staining were performed by Servicebio, China. The analysis was performed using the ImageJ software (National Institutes of Health and the Laboratory for Optical and Computational Instrumentation).

### Fibroblastic colony-forming units (CFU-F) assay

At the time of euthanasia, we collected the bone marrow from the femoral cavities of the mice by flushing it with a 25-gauge needle and determined the cell numbers with Zap-OGLOBIN (Coulter Corp.) after removing red blood cells. As reported, the number of CFU-Fs in isolated mouse bone marrow cells was determined by co-culturing with irradiated guinea pig marrow cells [32].

To achieve the guinea pig marrow as feeder cells, we obtained the bone marrow cells from the femur of 2-month-old female Hartley guinea pigs (Guangdong Medical Laboratory Animal Center, Guangzhou, China) by flushing with a 22-gauge needle and then resuspended the cells. The guinea pig marrow cells were irritated with a cobalt-57 source for 50 min at 1.2 Gy/min. All cells were resuspended in α-MEM medium with 20% FBS (PAN-Biotech), counted, and cultured at 2.5 × 10^6^ cells per well of a six-well plate.

For the assay of CFU-F number, we plated 0.1 × 10^5^, 0.5 × 10^5^, or 1 × 10^5^ bone marrow cells from the femur of mice into a well in a six-well plate, culturing with α-MEM (Gibco) supplemented with 2 mM glutamine, 1× penicillin/streptomycin (Invitrogen), and 20% FBS (PAN-Biotech). After 2–3 h of adhesion, we removed the unattached cells and added 2.5 × 10^6^ irradiated guinea pig feeder cells to the medium of the adherent cultures just after washing with PBS. On day 14, the cells were fixed with 4% PFA and stained with 0.5% crystal violet. Only the colonies that contained 50 or more cells were counted.

### Isolation of mesenchymal stromal cells

Mesenchymal stromal cells (MSC) were isolated as reported [33]. In brief, the mice were anesthetized and prepared with 70% ethanol to avoid bacterial contamination. The femur was dissected on a clean bench. The bones were stored in DMEM (low glucose, Gibco) supplemented with 1× penicillin/streptomycin on ice. The ends of the femur were cut, and the bone marrow cells were flushed with a 25-gauge needle. The cell suspension was filtered through a 70-mm filter mesh. The yield and viability of cells were determined by Trypan blue exclusion and counting on a hemocytometer. The cells were plated into six-well plates with DMEM (low glucose, Gibco) supplemented with 1× penicillin/streptomycin (Invitrogen) and 15% FBS (PAN-Biotech) at 37 °C in a 5% CO_2_ humidified incubator. Three hours later, remove the non-adherent cells that accumulate on the surface of the dish by replacing the medium and left the cells at 37 °C in a 5% CO_2_ humidified incubator. Replace the medium every 8 h until 72 h since the first medium replacement. After that, the medium was replaced every 3–4 days. Passage the cells to achieve the 1st passage until the confluence reaches 70%. Change the medium every 3–4 days until the confluence was reached. Passage these cells again to achieve the purified MSC.

### Growth rate test of MSC

4 × 10^5^ MSC of the 2nd passage were put into a well of the 6-well plate. Change the medium every 2 days and passage the cells on the 4th day. During passaging, the cells were counted by blood counting chamber, and we placed 4 × 10^5^ MSC of the 3rd passage in another well. Replace the medium every 2 days and passage the cells on the 8th day. Cell counting was performed again to analyze the MSC growth rate.

### Multilineage differentiation

After 5 generations, the isolated MSC was then cultured for 3 weeks in either adipogenic or osteogenic differentiation media. Adipogenic medium was DMEM (high glucose, Gibco) supplemented with 100 nM dexamethasone (Sigma), 0.5 mM isobutyl-methylxanthine (Sigma), 0.2 mM indomethacin (Sigma), 10 μg/ml insulin (Sigma), 1× penicillin/streptomycin (Invitrogen), and 10% FBS (PAN-Biotech). Osteogenic medium was DMEM (low glucose, Gibco) supplemented with 100 nM dexamethasone (Sigma), 10 mM β-glycerolphosphate (Sigma), 10 μg/ml ascorbic acid (Sigma), 1× penicillin/streptomycin (Invitrogen), and 20% FBS (PAN-Biotech). Cells were fed every 3 days. Four weeks later, the cells were fixed and then stained with Oil Red O and Alizarin Red S for adipocytes and osteoblasts, respectively.

### Statistical analysis

SPSS 21.0 (SPSS Inc.) and GraphPad Prism 7.0 (GraphPad Software) were used for statistical analysis. All data are presented as the mean ± SD. Statistical significance was estimated using Student’s *t* test. A two-sided *P* value < 0.05 was considered to be statistically significant. The level of significance is indicated in the figures as *P < 0.05, **P < 0.01, and ***P < 0.001.

## Results

### Erythropoiesis is defective in cancer cachexia

**Fig. 1:**
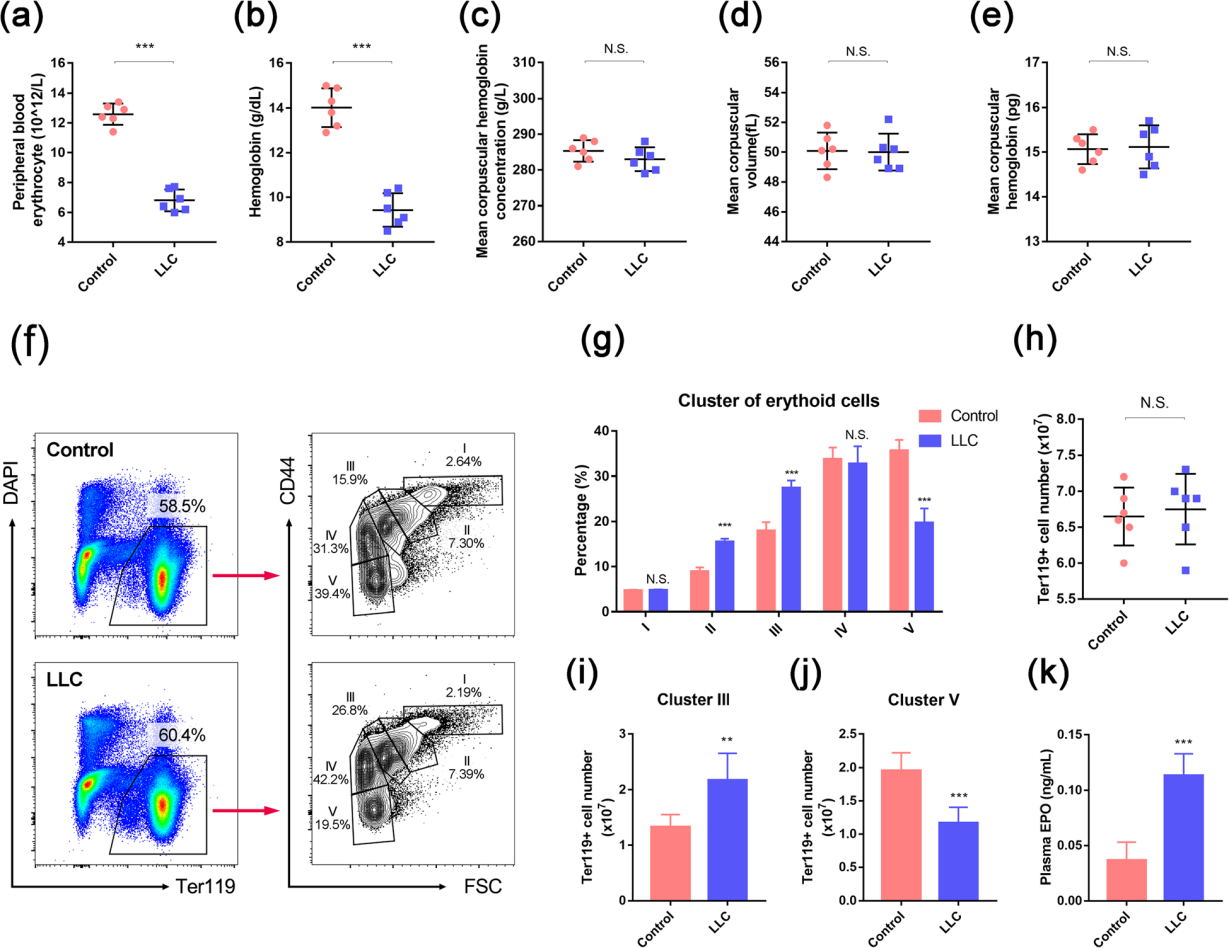
Obstruction of erythroid differentiation in CRA mouse model. **a**–**e** Red blood cell (RBC) level, hemoglobin level, mean corpuscular hemoglobin concentration (MCHC), mean corpuscular volume (MCV), and mean corpuscular hemoglobin (MCH) in control and LLC-bearing mice at 3 weeks after tumor cell inoculation (*n* = 6/group). **f** Representative flow cytometry profiles of erythroid cells in control and LLC-bearing mice. Viable (impermeable to DAPI) Ter119^+^ cells were gated and further analyzed with respect to FSC and CD44 surface expression, which allowed the subgrouping of erythroid cells. Clusters I–V, representing proerythroblasts (I), basophilic erythroblasts (II), polychromatic erythroblasts (III), orthochromatic erythroblasts/immature reticulocytes (IV), and mature red cells (V), were gated, and their percentages are shown. **g** Percentages of different erythroid cell clusters among Ter119^+^ cells. **h** The number of Ter119^+^ cells in the bone marrow of control and LLC-bearing mice. **i**, **j** The number of cluster III and cluster V cells in Ter119^+^ cells from the bone marrow of a single femur was calculated. **k** The concentration of plasma erythropoietin (EPO) in control and LLC-bearing mice. Data are presented as the means ± SD of three independent experiments. **P < 0.01, ***P < 0.001; N.S., no significance; assessed by Student’s *t* test

To detect the features of CRA, we constructed a tumor-bearing model through subcutaneous injection of the LLC cell line into the flanks of mice (*n* = 6/group) (Fig. S1a-b). After 21 days, we examined the routine blood parameters and found that the LLC-bearing mice developed normocytic anemia: hemoglobin (Hb) and RBC levels decreased significantly, while the mean corpuscular hemoglobin concentration (MCHC), mean corpuscular volume (MCV), and mean corpuscular hemoglobin (MCH) remained normal (Fig. 1a–e). We analyzed the populations of erythroid cells in various stages by sorting the cells for the expression of Ter119 (expressed on committed erythroid cells) and CD44 (progressively reduced during erythroid differentiation) [9]. In this way, the Ter119^+^ cells in the bone marrow could be divided into five clusters: pro-E (I), basophilic erythroblasts (II), polychromatic erythroblasts (III), orthochromatic erythroblasts, immature reticulocytes (IV), and mature red cells (V) (Fig. 1f, g). The total number of Ter119^+^ cells remained unchanged (Fig. 1h). However, the proportion and number of cells in cluster III were increased in LLC-bearing mice (Fig. 1g, i), whereas the proportion and number of cells in cluster V were decreased (Fig. 1g, j), suggesting that erythropoiesis in these mice was blocked between the stages of polychromatic erythroblasts and mature red cells. These data indicated that erythropoiesis is suppressed during CRA and the late-stage of cell maturation. EPO is one of the most significant pro-erythropoietic cytokines. Anemia can be attributed to the insufficiency of unresponsiveness of EPO. However, in this model, EPO increased 4-fold during the development of CRA (Fig. 1k), which might be the feedback loop of anemia-induced hypoxia.

### The hematopoiesis is hindered in the bone marrow of CRA mice

**Fig. 2:**
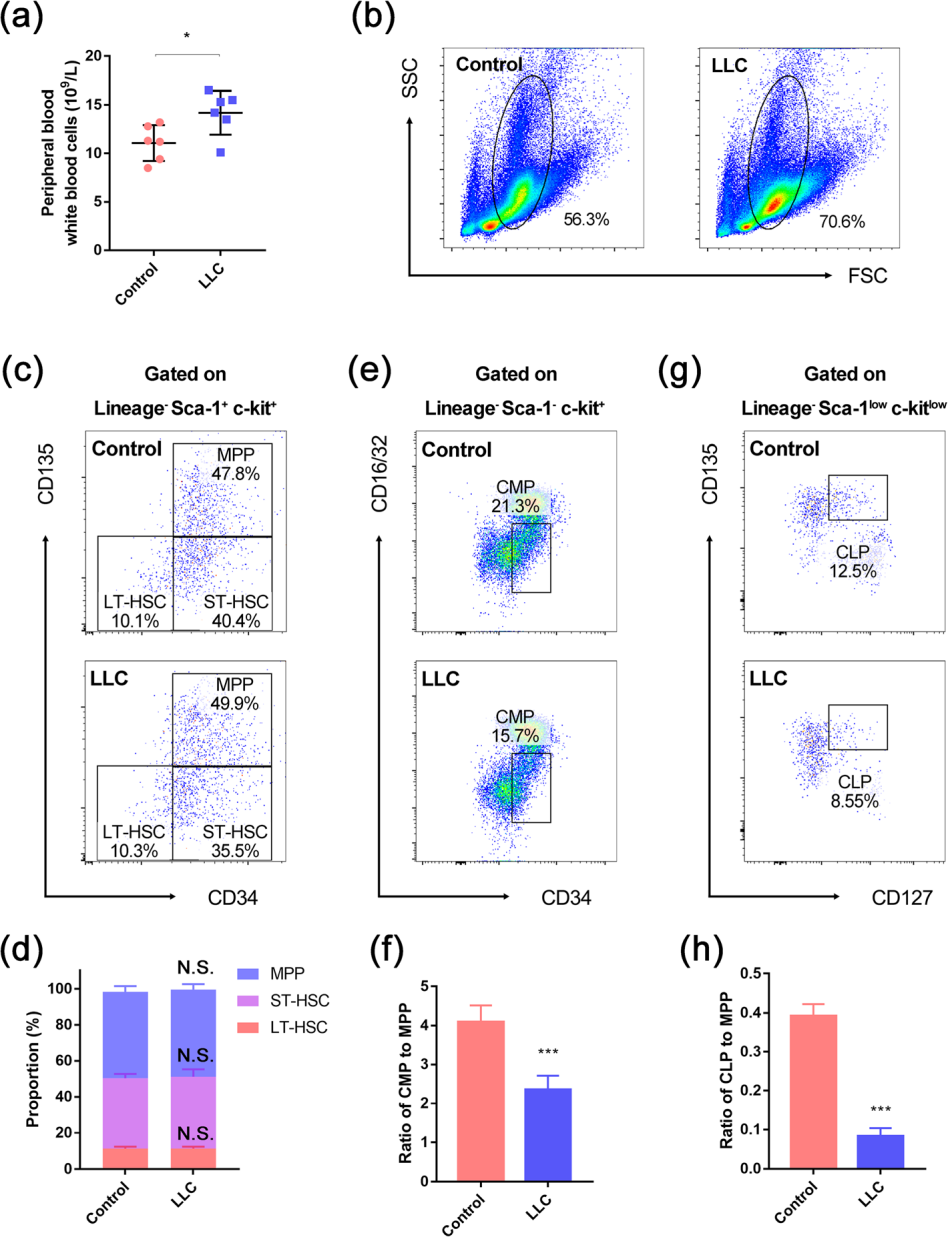
Differentiation of HSCs was suppressed during CRA. **a** The numbers of peripheral blood white blood cells in control and LLC-bearing mice. **b** Representative flow cytometric profiles of myeloid cells in the bone marrow. **c** Representative flow cytometric images of long-term/short-term hematopoietic stem cells (LT/ST-HSC) and multipotent progenitors (MPP) in the bone marrow of control and LLC-bearing mice. **d** The proportion of LT/ST-HSC and MPP in Lineage^−^ Sca1^+^ c-kit^+^ (LSK) cells of control and LLC-bearing mice. **e** Representative flow cytometric images of common myeloid progenitors (CMP) in the bone marrow of control and LLC-bearing mice. **f** The ratio of CMP to MPP in the bone marrow of contro and LLC-bearing micel (*n* = 6). **g** Representative flow cytometric images of common lymphoid progenitors (CLP) in the bone marrow of control and LLC-bearing mice. **h** The ratio of CLP to MPP in the bone marrow of control and LLC-bearing mice (n=6). Data are presented as the means ± SD of three independent experiments. *P < 0.05, ***P < 0.001; N.S., no significance; assessed by Student’s *t* test

Blood parameters also showed an increase in peripheral white blood cell numbers (Fig. 2a), indicating a stress reaction in the bone marrow. Flow cytometry revealed an increase in bone marrow cell number (Fig. S2a) and myeloid cell proportion (Fig. 2b and Fig. S2b). These results suggest that CRA is related not only to the maturation of erythrocytes but also to the whole hematopoietic process, so we performed flow cytometry to investigate the differentiation of the hematopoietic stem and progenitor cells. The results showed that the proportion of LT-HSC, ST-HSC, and MPP in the Lineage^−^ Sca1^+^ c-kit^+^ (LSK) cells all stayed unchanged (Fig. 2c, d), which indicated that the differentiation before HSC lost its multipotency was only influenced little. However, the ratio of CMP to MPP (Fig. 2e, f) and the ratio of CLP to MPP (Fig. 2g, h) were both decreased in the LLC-bearing mice. These results showed that, although the capability of LT/ST-HSC in generating the descendants was only impacted little, the differentiation of MPPs into the committed progenitors was dramatically impaired in the LLC-bearing mice, which indicated a hindered hematopoietic process.

### Osteoclastic bone resorption increases during CRA pathogenesis

**Fig. 3:**
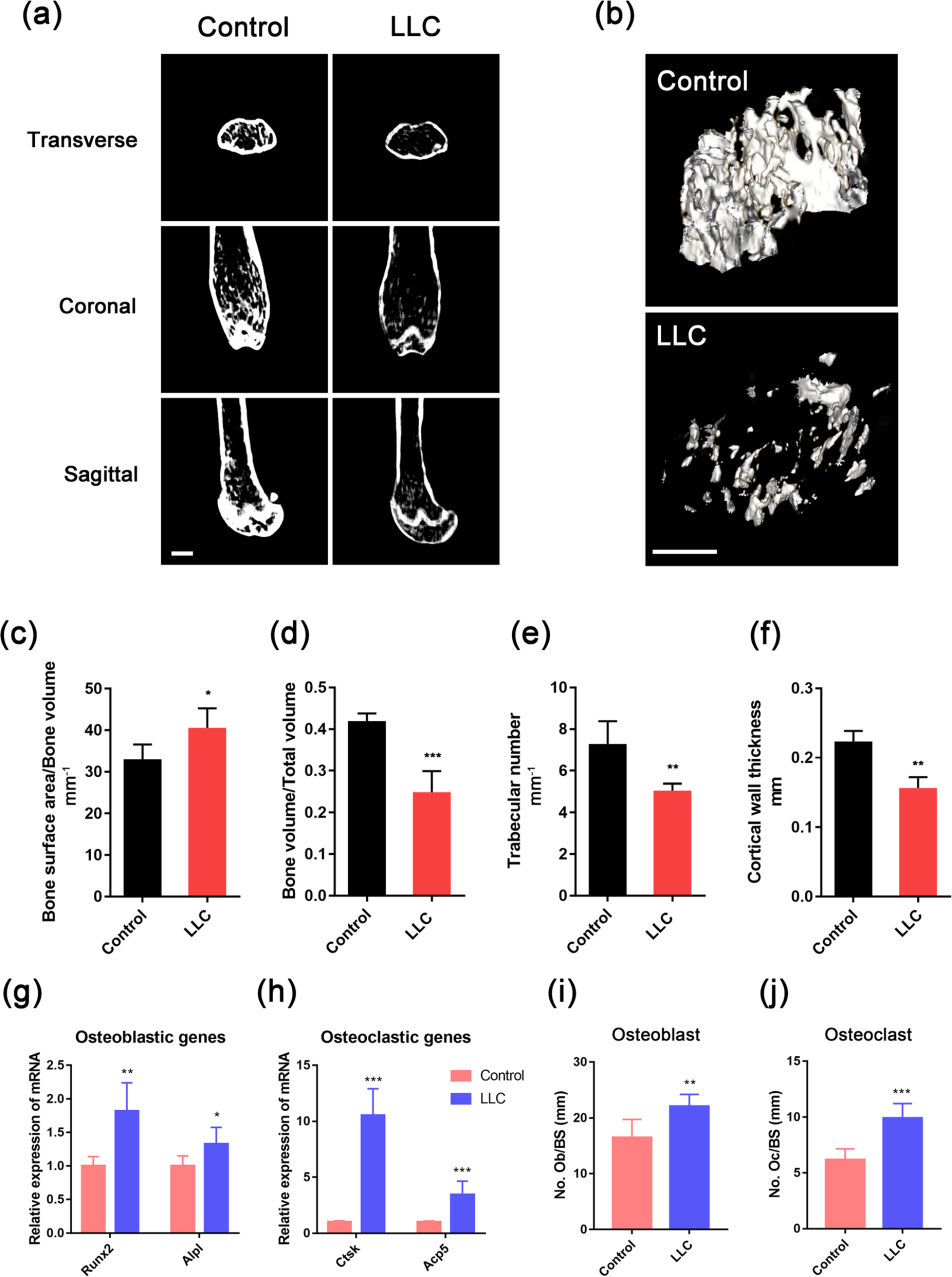
Osteoclastic bone resorption is increased during CRA. **a**, **b** Representative three-dimensional thickness maps from micro-computed tomography (micro-CT) scans of the trabecular bone from the distal femur metaphysis of control and LLC-bearing mice. Scale bar, 1000 μm. **c**–**f** Ratio of bone surface area to bone volume, ratio of bone volume to total volume, trabecular number, and cortical wall thickness were calculated. **g**, **h** The mRNA expression levels of osteoblastic and osteoclastic genes in control and LLC-bearing mice (*n* = 6/group). **i**, **j** The numbers of osteoblasts and osteoclasts in the trabecular bone from control and LLC-bearing mice were calculated (*n* = 6/group). Data are presented as the means ± SD of three independent experiments. *P < 0.05, **P < 0.01, ***P < 0.001; assessed by Student’s *t* test

During the dissection of the femurs, we were surprised to discover that the femurs from LLC-bearing mice were more fragile than those of control mice, suggesting that the bone had undergone an osteolytic process. Thus, we used micro-computed tomography (micro-CT) to determine the change in bone mass during CRA (Fig. 3a, b). The results showed that the cortical and trabecular bones of the femurs underwent a remarkable mass loss in LLC-bearing mice compared to control mice (Fig. 3a–f and Fig. S3a-c). To further determine the etiology of this cancer-related bone loss, we examined the mRNA expression of osteoblastic and osteoclastic genes in the femoral bone marrow. The results showed an increase in the expression of the osteoblastic genes, including runt-related transcription factor 2 (*Runx2*) and alkaline phosphatase liver/bone/kidney (*Alpl*), as well as the osteoclastic genes, including cathepsin K (*Ctsk*) and acid phosphatase 5 (*Acp5*), in LLC-bearing mice (Fig. 3g, h). The section staining of the femur sections with tartrate-resistant acid phosphatase (TRAP) kits and antibodies against Runx2 showed that the numbers of osteoblasts and osteoclasts were both increased in the trabecular bone (Fig. 3i, j and Fig. S4a-b). However, the elevation of osteoclastic activity was more intensive than that of the osteoblastic process (Fig. 3g–j and Fig. S4a-b), which indicated that the bone mass loss in CRA mice was attributed to the excessive osteoclastic activity.

### TGFβ affects the HSC niche in the bone marrow

**Fig. 4:**
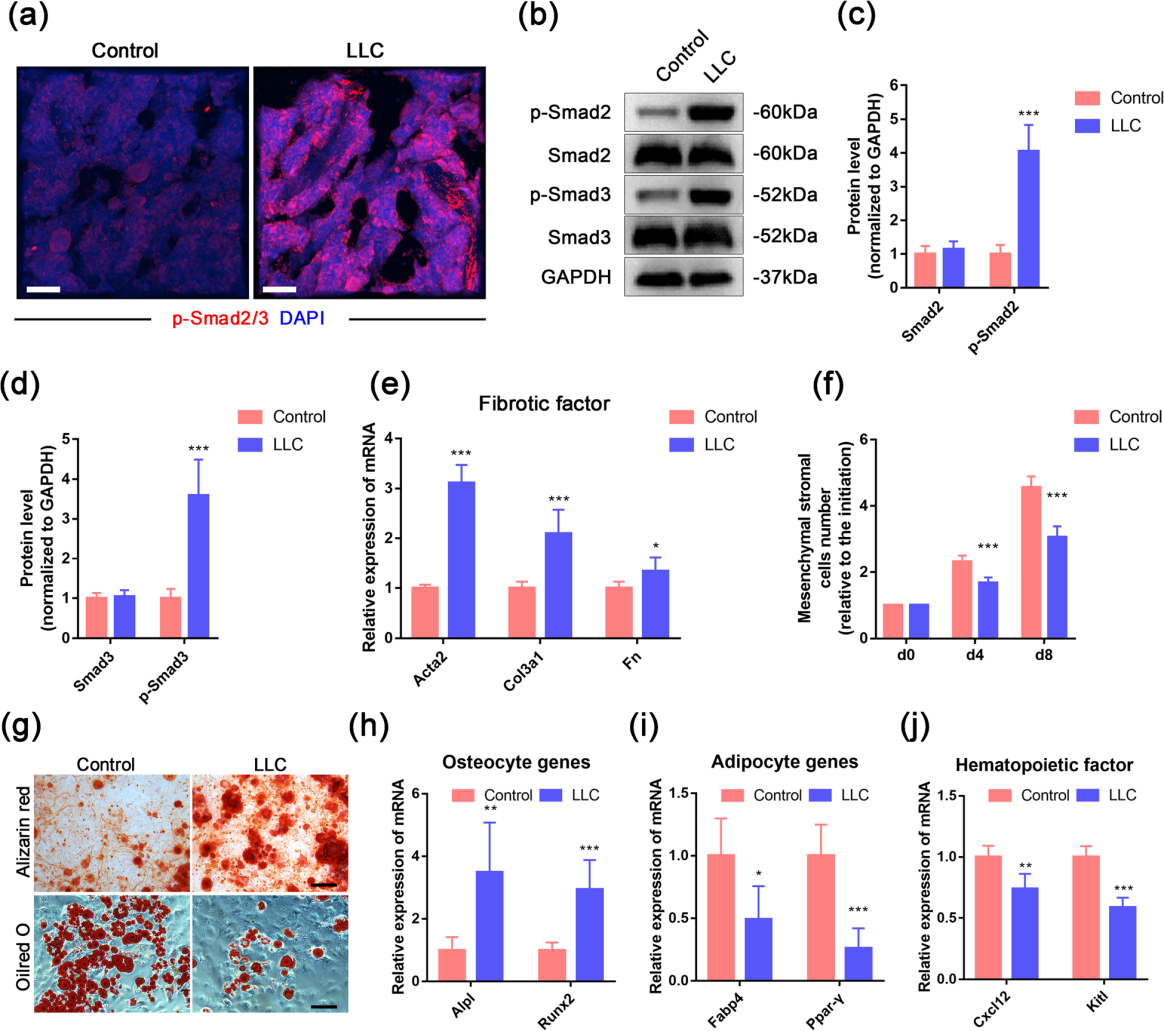
TGFβ deteriorated HSC niche by affecting MSC. **a** Representative confocal images show the expression of phosphorylated Smad2/3 in the trabecular bone of control and LLC-bearing mice. Scale bar, 50 μm. **b**–**d** Western blotting analysis and quantifications of phosphorylated Smad2/3 and total Smad2/3 proteins in the bone marrow from control and LLC-bearing mice. **e** mRNA levels of the fibrotic factors, *Acta2*, *Col3a1*, and *Fn*, in the bone marrow from control and LLC-bearing mice were analyzed by qPCR. **f** MSC growth rate was shown by cell number relative to the initiation (0d in the panel), which contains 4 × 10^5^ cells of the 2nd passage per well. **g** Panel shows lineage differentiation of isolated MSC into either osteoblast (Alizarin Red) or adipocytes (Oil Red). Scale bars, 100 μm. **h**, **i** The mRNA levels of osteoblastic genes, alkaline phosphatase liver/bone/kidney (*Alpl*) and runt-related transcription factor 2 (*Runx2*), and adipogenic genes, fatty acid-binding protein 4 (*Fabp4*) and peroxisome proliferator-activated receptors-γ (*Ppar-γ*), were analyzed by qPCR in differentiated bone marrow MSC. **j** The mRNA expression of the hematopoietic factors, *Cxcl12* and *KitL*, in control and LLC-bearing mice. *P < 0.05, **P < 0.01, ***P < 0.001; assessed by Student’s *t* test

Studies have demonstrated that the bone matrix is the largest source of TGFβ in the bone marrow niche, and during bone resorption, TGFβ can be released from the bone matrix [22]. TGFβ has so many target cells that excessive TGFβ would trigger multiple pathological changes. Therefore, to define whether there was a TGFβ signaling activation, we performed immunofluorescence analysis to investigate the phosphorylation levels of Smad2 and Smad3 in the bone marrow of CRA mice. The results showed that the level of p-Smad2/3 was elevated in the bone marrow of CRA mice (Fig. 4a), and the Western blotting of bone marrow cells also revealed the increased phosphorylation levels of Smad2 and Smad3 after LLC-bearing (Fig. 4b–d), indicating that the TGFβ signaling was activated in the bone marrow during CRA. The raised active TGFβ1 level in the serum of the mouse model has further confirmed our hypothesis (Fig. S5a). As TGFβ is a well-known pro-fibrotic cytokine related to bone marrow fibrosis [34], we further detected the markers of fibrosis in bone marrow, including the myofibroblast marker, actin alpha 2 (*Acta2*), and the fiber component, collagen type III alpha 1 (*Col3a1*) and fibronectin (*Fn*). As evidenced by the elevated expression of *Acta2*, *Col3a1*, and *Fn* in the bone marrow of LLC-bearing mice (Fig. 4e), the qPCR data suggested that the bone marrow underwent a fibrotic switch during CRA. Since mesenchymal stromal cells (MSC) are proven to be one of the main sources of myofibroblasts [29, 35], we isolated and analyzed the MSC in the bone marrow. The cell growth rate showed a significant decline in the CRA mice (Fig. 4f), and the capacity of multipotent differentiation of MSC was also altered (Fig. 4g–i). Osteogenic and adipogenic potentials were analyzed by cytochemistry staining and qPCR detection of differentiated cell markers. The osteogenic potential was elevated in LLC-bearing mice (Fig. 4g, h), while the adipogenic capacity was declined (Fig. 4g, i). These results were consistent with the pro-osteogenic effect of TGFβ. Meanwhile, because MSC is also an important HSC niche component and a source of hematopoietic cytokines [10, 36], we further tested the expression of *Cxcl12* and *Kitl* in bone marrow cells. Interestingly, both of the hematopoietic factors showed a dramatic decline (Fig. 4j). These results indicated that there was a deterioration in the HSC niche of CRA mice, which somehow impaired the differentiation of hematopoietic cells.

### SB505124 treatment ameliorates anemia in the CRA model

**Fig. 5:**
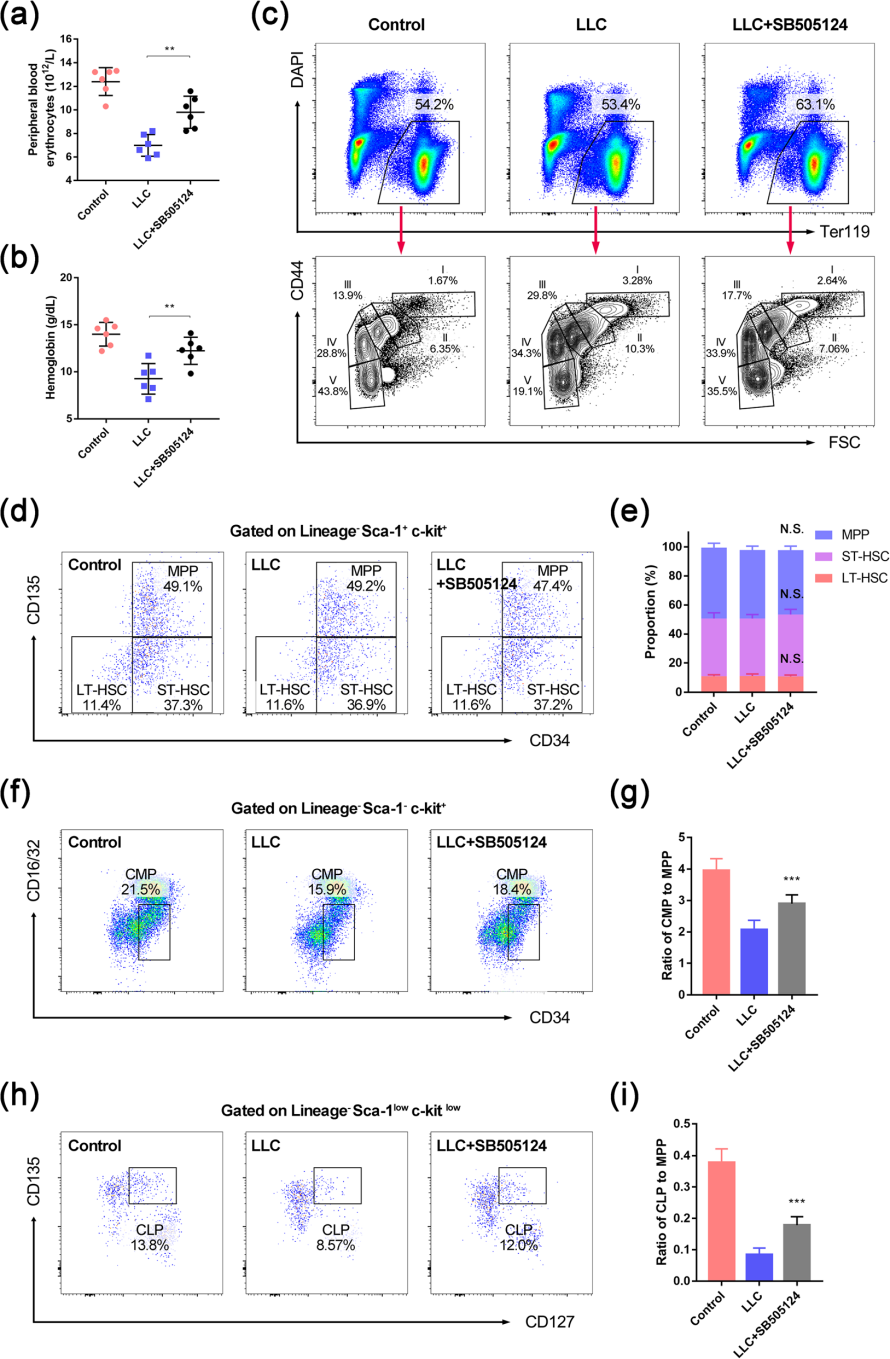
SB505124 rescued CRA symptoms in both the peripheral blood and bone marrow. **a** The peripheral blood erythrocyte level and **b** hemoglobin level in control (PBS-treated), LLC-bearing mice (LLC), and SB505124-treated LLC-bearing mice (LLC+SB505124) were calculated (*n* = 6/group). **c** Representative flow cytometry profiles of erythroid cells in control, LLC, and LLC+SB505124 mice. DAPI^−^ viable Ter119^+^ cells were gated and analyzed with FSC and CD44 surface expression to demonstrate the clusters of erythroid cells. Clusters I–V represented the proerythroblasts (I), basophilic erythroblasts (II), polychromatic erythroblasts (III), orthochromatic erythroblasts/immature reticulocytes (IV), and mature red cells (V). Their percentages are shown in the representative profiles. **d** Representative flow cytometric images of LT/ST-HSC and MPP in the bone marrow of control, LLC, and LLC+SB505124 mice. **e** The proportion of LT/ST-HSC and MPP in LSK cells of control, LLC, and LLC+SB505124 mice (*n* = 6). **f** Representative flow cytometric images of CMP in the bone marrow of control, LLC, and LLC+SB505124 mice. **g** The ratio of CMP to MPP in the bone marrow of control, LLC, and LLC+SB505124 mice (*n* = 6). **h** Representative flow cytometric images of CLP in the bone marrow of control, LLC, and LLC+SB505124 mice. **i** The ratio of CLP to MPP in the bone marrow of control, LLC, and LLC+SB505124 mice (*n* = 6). Data are presented as the means ± SD of three independent experiments. **P < 0.01, ***P < 0.001; N.S., no significance; assessed by Student’s *t* test

The inhibitor of TGFβ signaling, SB505124, was previously shown to be effective in ameliorating various orthopedic diseases [19, 23]. Here, we examined whether SB505124 could mitigate the CRA by blocking the TGFβ signaling. Mice were intraperitoneally injected with SB505124 (5 mg/kg, daily) starting on day 7 after tumor cell implantation (Fig. S6a). To determine whether it was effective, we first analyzed the erythropoiesis in the SB505124-treated CRA model. Interestingly, we found that the reduction of both peripheral blood erythrocytes and hemoglobin were both ameliorated by SB505124 treatment (Fig. 5a, b). Furthermore, the cluster distribution of erythroid cells was recovered to a certain extent (Fig. 5c): in SB505124-treated CRA mice, the proportion and the number of cells in cluster III were decreased (Fig. 5c and Fig. S6b-c), while those in cluster V were significantly increased (Fig. 5c and Fig. S6d-e). In addition, the number of white blood cells in the peripheral blood decreased in the treated group (Fig. S6f), as did the proportion of myeloid cells (Fig. S6g). And interestingly, after treated with SB505124, the ratios between CMPs/CLPs and HSCs were also increased, without influencing the proportion of LT/ST-HSC and MPP (Fig. 5d–i). Collectively, these results indicated that SB505124 could improve the erythropoiesis in the LLC-bearing mice, as well as the hindered differentiation of hematopoietic progenitors.

### SB505124 attenuates the HSC niche deterioration

**Fig. 6:**
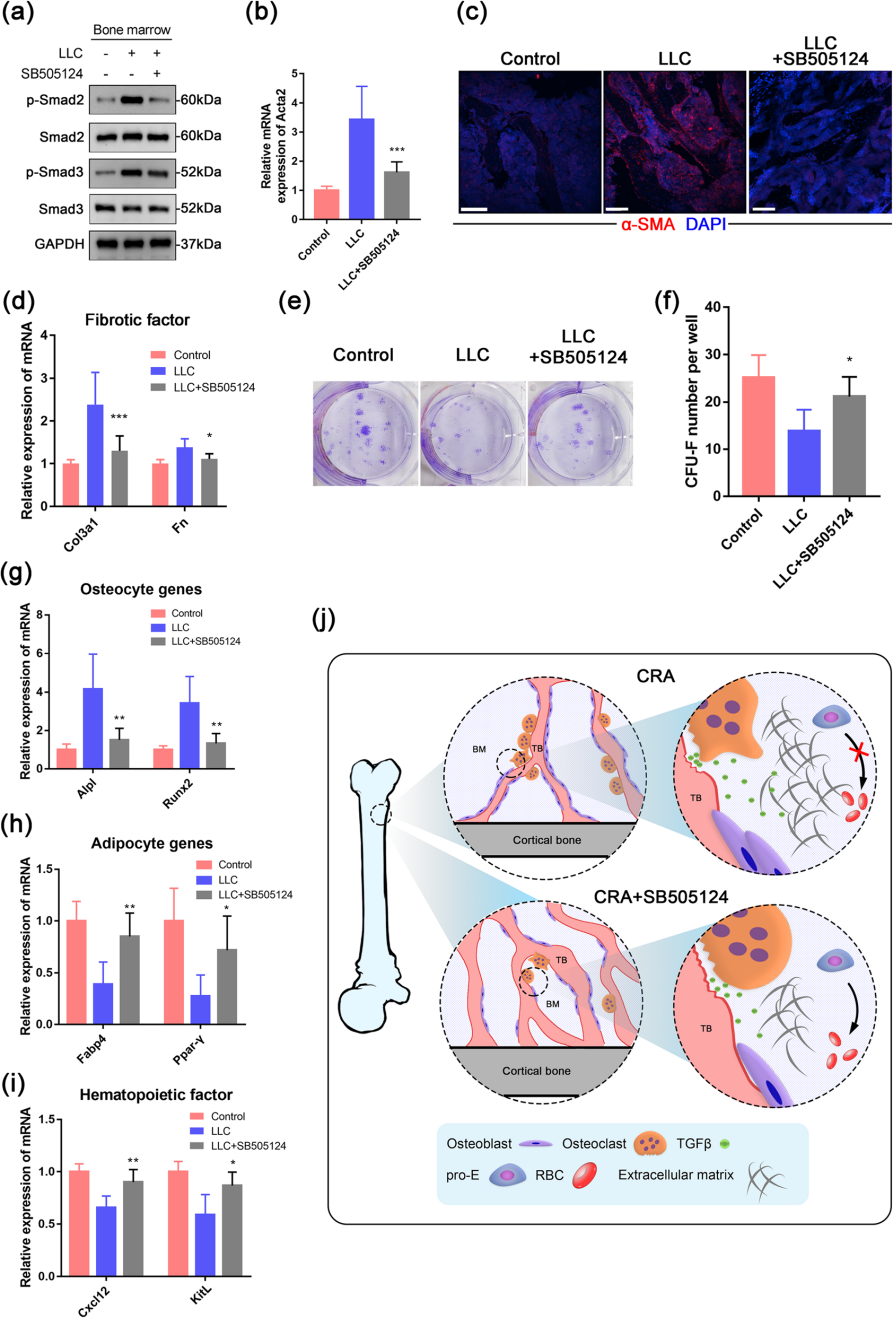
Inhibition of the TGFβ pathway rescues the HSC niche deterioration. **a** Western blotting analysis of phosphorylated Smad2/3 and total Smad2/3 proteins in the bone marrow of control, LLC, and LLC+SB505124 mice. **b** mRNA expression of *Acta2* in the bone marrow of control, LLC, and LLC+SB505124 mice. **c** Representative confocal images show the expression of α-SMA protein in the trabecular bone of control, LLC, and LLC+SB505124 mice. Scale bar, 100 μm. **d** mRNA expression of the fibrotic factors, *Col3a1* and *Fn*, in the bone marrow of control, LLC, and LLC+SB505124 mice. **e**, **f** CFU-F assays and quantification from the bone marrow of control, LLC, and LLC+SB505124 mice. Representative images of CFU-Fs stained with crystal violet. **g**, **h** qPCR detection of osteoblastic genes and adipogenic markers in differentiated MSC. **i** The mRNA expression of the hematopoietic factors, *Cxcl12* and *KitL*, in the bone marrow of control, LLC, and LLC+SB505124 mice. **j** During CRA, the osteoclastic process released excessive TGFβ and activated the TGFβ pathway in the bone marrow, which induced a deterioration of the HSC niche and the blockage of hematopoiesis. By inhibiting the TGFβ signaling pathway with SB505124, the HSC niche and hematopoiesis were restored, and the CRA symptoms also showed relieved. *P < 0.05, **P < 0.01, ***P < 0.001; assessed by Student’s t test

To further determine the effect of SB505124 on the hematopoietic niche, we firstly analyzed the level of p-Smad2/3 to investigate the level of TGFβ signaling, which confirmed the suppression of TGFβ signaling in the bone marrow of SB505124-treated mice (Fig. 6a and Fig. S7a-b). Furthermore, qPCR and immunostaining were performed and showed that the expression of *Acta2*, as well as the level of *Col3a1* and *Fn*, were all significantly downregulated after the treatment (Fig. 6b–d). As for the condition of MSC, we applied Nestin-GFP transgene mice to sort the Nestin-GFP^+^ bone marrow MSC [36, 37] and analyzed the mRNA expression of TGFβ target genes. As expected, the activation of TGFβ signaling in MSC was shown by the increased target genes level (Fig. S7c). Meanwhile, we also determined the proliferative capacity of the MSC with applied fibroblastic colony-forming units (CFU-F) assay, in which the number of CFU-Fs was restored in the SB505124-treated group (Fig. 6e, f). Moreover, the MSC derived from the treated mice showed a decreased osteogenic capacity (Fig. 6g) and an enhancement in adipogenesis (Fig. 6h), which might be attributed to the suppressed pro-osteoblastic effect of TGFβ. In addition, the mRNA levels of hematopoietic factors were increased in the SB505124-treated group (Fig. 6i), indicating the alleviation of deteriorated HSC niche. And even the osteoclastic bone loss was restored after the treatment (Fig. S8a-f). Together, these results suggested that targeting TGFβ signaling with SB505124 can restore the function of MSC, the important HSC niche component, and thus improve the hematopoietic microenvironment.

## Discussion

CRA is a common complication of cancer patients with cachexia. As reported, more than 30% of cancer patients at diagnosis show anemic symptoms [4, 5]. CRA is usually associated with cancer-related fatigue and overall impairment in quality of life, and it is considered to be an independent adverse prognostic factor in cancer patients [38]. The treatments for CRA usually involve red blood cell transfusion, iron therapy, and erythropoietin supplementation. However, none of these treatments is specific to the etiology of the disease [39, 40]. In this study, we generated a CRA mouse model with LLC administration and found that the changes in osteoclastic bone resorption were associated with CRA. Activation of the TGFβ signaling pathway in the bone marrow during LLC-induced osteoclastic bone resorption inhibited the differentiation of the erythroid lineage and induced deterioration of the HSC niche in the bone marrow to further impair hematopoiesis. An inhibitor of the TGFβ signaling pathway, SB505124, was found to attenuate this deterioration to the bone marrow niche and relieve the hematopoietic disorders of bone marrow.

Along with the reduction of RBC in the peripheral blood, we observed that erythropoiesis was blocked to generate mature RBC in the bone marrow of the CRA model. Given that circulating RBC are the progeny of HSC, which are regulated by the HSC niche [41, 42], dysfunction of the niche will lead to disordered erythropoiesis with impaired erythroid progenitor cell differentiation and maturation, yielding anemic symptoms [10]. Within the bone marrow parenchyma, two indispensable components, the endosteum and the stroma, contribute to maintaining the homeostasis of HSC [43], and they exist in a dynamic equilibrium between the cells and matrix. The endosteum provides mechanical protection and cytokines for bone marrow cells and is thus intrinsically linked to hematopoiesis [44]. Osteoblasts and osteoclasts, the two main cell types in the endosteum, orchestrate the balance between bone modeling and remodeling and thereby modulate the self-renewal, proliferation, and differentiation of HSC [45]. Visnjic et al. showed that selective depletion of osteoblasts leads to a reduction in HSC number, whereas an increase in osteoblast number augments the HSC pool in the bone marrow [46]. Moreover, osteoclast inhibition increases HSC mobilization in response to G-CSF and reduces the retention of primitive HSC [47]. Stimulation of osteoclast activity induces the expansion of hematopoietic progenitor cells, which is mediated by the production of some components of the HSC niche, such as SDF-1 or SCF [48]. It has also been proposed that osteoclasts promote the formation of the HSC niche via crosstalk with osteoblasts [49]. In the present study, we observed a significant increase in osteoclasts of CRA mice. Despite the pro-osteoblastic function of TGFβ, we noted only a slight increase in the osteoblast number. As osteoblasts are MSC progeny, the lack of osteoblast activity might be attributed to the change in MSC.

In addition to the pathophysiological changes in the endosteum, we also observed disturbance of the bone marrow stroma in LLC-bearing mice, which exhibited upregulation of fibrotic genes and impairment of MSC. In the normal physiological state, the stroma matrix physically supports HSC, and the MSC secretes numerous paracrine factors, such as SDF-1, SCF, and angiogenin [50]. These factors are quite important for maintaining the homeostasis of the hematopoietic niche and regulating the fate of HSC [11, 51]. In the pathogenesis of primary myelofibrosis, however, stromal cells can act as a source of myofibroblasts and induce the deposition of extracellular matrix [29]. Therefore, disruption of the stroma in diseased states can greatly affect hematopoietic homeostasis [52, 53]. In the present work, we observed the upregulation of *Acta2* (indicating the expansion of myofibroblasts) and the fibrotic genes *Col3a1* and *Fn* in the bone marrow of LLC-bearing mice. Interestingly, Decker et al. previously reported that during myelofibrosis, mice exhibit leukocytosis, and the bone marrow myeloid cell proportion expands [29]. The HSC number was found to be increased in myelofibrosis mice, while bone marrow cellularity did not increase accordingly [29]. Similarly, in our CRA model, the white blood cells in the peripheral blood and the myeloid lineage both increased. Non-conformity between the number of HSC and bone marrow cells was also observed, consistent with previous work. Meanwhile, the ratio of MPP/CLP to MPP was decreased in the CRA mice, further demonstrated the hindered hematopoiesis in the mouse model. Because many studies have demonstrated that hematopoiesis is disordered during bone marrow fibrosis, our results indicate that fibrotic changes in stromal cells might be involved in the pathogenesis of CRA. Future work is needed to clarify the underlying mechanism.

TGFβ is a ubiquitous cytokine that plays roles in physiological functioning throughout the lifespan [54]. In LLC-bearing mice, evidence has indicated that the TGFβ signaling pathway is activated in the bone marrow. As TGFβ receptors exist on various kinds of cells, the elevation of TGFβ levels plays a critical role in numerous physiological and pathological processes [55]. It is well-proven that TGFβ signaling is highly involved in the direct regulation of hematopoietic stem and progenitor cells [56]. For one thing, in some hematologic malignancies-induced bone marrow failure, TGFβ signaling is activated in hematopoietic progenitors [57, 58], and over-activation of the pathway in vitro can dramatically suppress the maturation of these cells [59]. Another pharmacologic inhibition of the pathway has also been demonstrated to restore the hindered hematopoiesis under pathological states in vitro and vivo [34, 60, 61], revealing the direct regulatory function of TGFβ in the cell fate of hematopoietic progenitors. Moreover, TGFβ plays a critical role in regulating the formation of erythrocytes [16]. It works synergistically with EPO to force the differentiation of CFU-E to more mature stages and it can also block erythropoiesis by suppressing the mitotic activity of CFU-E [16]. However, TGFβ receptors are also expressed on the cells in the HSC niche [62]. On the one hand, TGFβ has been shown to be a predominant cytokine involved in inducing the expansion of pro-fibrotic cells and the deposition of extracellular matrix [63]. It is responsible for fibrosis in multiple organs [64, 65]. On the other hand, the osteoblastic process, a special type of extracellular matrix deposition, can also be induced by TGFβ. The disturbance of TGFβ has proven to be involved in the pathogenesis of hyperostosis and osteoarthritis [23, 24]. However, only a minority of studies have demonstrated the function of TGFβ in regulating the niche cells. Therefore, rather than paying attention to the direct regulatory effect of TGFβ on hematopoietic cells as previously reported, in our study, we highlighted the reduction of MSC and the increased fiber deposition under TGFβ over-activation, in order to show the significance of TGFβ in hematopoiesis from the perspective of niche maintenance.

In our study, SB505124 was found to be effective in rescuing symptoms during CRA. SB505124 is a small molecule inhibitor of the TGFβ type I receptor serine/threonine kinase known as activin receptor-like kinase (ALK) [66]. DaCosta et al. found that SB505124 selectively and concentration-dependently inhibits ALK4-, ALK5-, and ALK7-induced Smad2 and Smad3 signaling but does not alter ALK1-, ALK2-, ALK3-, or ALK6-induced signaling [67]. A previous study suggested that SB431542-induced suppression of TGFβ signaling at an early stage of CD31^+^CD34^+^ progenitor differentiation could induce the generation of erythroid cells [68]. Moreover, SB431542 significantly increased the number of erythroblasts in myelofibrosis patients, indicating that treatment with an ALK inhibitor could potentially improve hematopoiesis under pathological conditions [69]. Given that SB505124 is three to five times more potent than SB431542 [67], we supposed that it might have a more powerful effect on rescuing erythropoiesis defects in our mouse model.

Indeed, we observed that SB505124 significantly rescued erythrocyte reduction, ameliorated the hindered hematopoiesis, and improved the HSC niche in the bone marrow. Our results suggest that the TGFβ signaling pathway could be targeted to restore the HSC niche and rescue CRA. Several TGFβ pathway inhibitors are currently under clinical trials and have shown acceptable safety, tolerability, and efficacy for slowing the progression of solid tumors and myelodysplastic syndrome. These include vactosertib (phase I) [70], galunisertib (phase II) [71], and pirfenidone (phase III) [72]. These details, combined with our present novel findings in the LLC-bearing mouse model, suggest that SB505124 is a safe and effective treatment that could be developed for CRA and potentially other cancer-related disorders.

## Conclusion

Our results indicated that osteolytic bone remodeling releases TGFβ and activates the pathway during CRA, along with deteriorating the HSC niche and seriously hindering hematopoiesis. The TGFβ signaling pathway inhibitor SB505124 can significantly restore the HSC niche, rescue hematopoiesis, and alleviate the symptoms of CRA in our mouse model (Fig. 6j).

## Supplementary Information

**Additional file 1:** **Fig. S1.** General characteristics of LLC mice model. **a** The tumor volume of tumor-bearing mice since LLC implantation (*n*=6). **b** Survival probability of control and LLC-bearing mice over time since LLC implantation (*n*=20). Data are presented as the means ± SD. **Fig. S2.** Hematopoiesis was influenced in bone marrow of CRA mice. **a** Cells number in bone marrow of control and LLC-bearing mice (*n*=6). **b** The percentage of myeloid cells in bone marrow of control and LLC-bearing mice (n=6). Data are presented as the means ± SD. *P<0.05, ***P<0.001; assessed by Student’s *t* test. **Fig. S3** LLC-bearing mice exhibited imbalanced bone remodeling. **a-c** Micro-computed tomography (micro-CT) analysis of trabecular spacing, trabecular pattern factor and trabecular thickness of trabecular bone from the distal femur metaphyses of controls and LLC-bearing mice. Data are presented as the means ± SD of three independent experiments. *P<0.05, **P<0.01; assessed by Student’s *t* test. **Fig. S4** The number of osteoclasts and osteoblasts was increased in cancer cachexia mice. **a** TRAP stain showing the osteoclasts in trabecular bone from control and LLC-bearing mice. Scale bar, 100 μm. **b** Immunofluorescence staining of Runx2 showing the osteoblasts in trabecular bone from control and LLC-bearing mice. Scale bar, 100 μm. **Fig. S5** Active form of TGFβ1 was increased in CRA mice. **a** The concentration of active TGFβ1 in the serum of controls and LLC-bearing mice. Data are presented as the means ± SD of three independent experiments. ***P<0.001; assessed by Student’s *t* test. **Fig. S6** SB505124 alleviated the hindered hematopoiesis in LLC-bearing mice. **a** Experimental design illustrating subcutaneous injection of DMSO or SB505124 (5 mg/day/kg) into control and LLC-bearing mice (*n*=6/group). **b-e** The percentage and number of Ter119+ cells in cluster III and cluster V in bone marrow of control, LLC and LLC+SB505124 mice. **f** The number of white blood cells in peripheral blood of control, LLC and LLC+SB505124 mice. **g** The percentage of myeloid cells in bone marrow of control, LLC and LLC+SB505124 mice. Data are presented as the means ± SD of three independent experiments. *P<0.05, **P<0.01, ***P < 0.001; assessed by Student’s *t* test. **Fig. S7** SB505124 inhibited TGFβ signaling activation in bone marrow and MSC. **a-b** Quantifications of the Western blotting of phosphorylated Smad2/3 and total Smad2/3 proteins in bone marrow of control, LLC and LLC+SB505124 mice (n=6/group). **c** mRNA expression of TGFβ target genes in MSC of control, LLC and LLC+SB505124 mice (n=6/group). Data are presented as the means ± SD of three independent experiments. *P<0.05; **P<0.01; ***P<0.001; assessed by Student’s *t* test. **Fig. S8** SB505124 relieved the osteolytic process of LLC-bearing mice. **a** The mRNA levels of osteoclastic genes in bone marrow of control, LLC and LLC+SB505124 mice (n=6/group). **b** Representative three-dimensional thickness maps from micro-CT scans of trabecular bone from the distal femur metaphysis of control, LLC and LLC+SB505124 mice. Scale bar, 1000 μm. **c-f** Ratio of bone surface area to bone volume, ratio of bone volume to total volume, trabecular number, and cortical wall thickness of control, LLC and LLC+SB505124 mice (*n*=3/group). Data are presented as the means ± SD of three independent experiments. *P<0.05; assessed by Student’s *t* test. **Supplemental Table S1.** Primer used to amplify the mouse transcripts during PCR. **Supplemental Table S2.** Primary and secondary antibodies.

## Data Availability

The datasets used and/or analyzed during the current study are available from the corresponding author on reasonable request.

## Abbreviations

CRA: Cancer-related anemia
LLC: Lewis lung carcinoma
TGFβ: Transforming growth factor β
Hb: Hemoglobin
HSC: Hematopoietic stem cells
MSC: Mesenchymal stromal cells
RBC: Red blood cells
Pro-E: Proerythroblasts
BFU-E: Erythroid burst-forming units
CFU-E: Erythroid colony-forming units
CFU-F: Fibroblastic colony-forming units
EPO: Erythropoietin
Micro-CT: Micro-computed tomography
PBS: Phosphate-buffered saline
MCHC: Mean corpuscular hemoglobin concentration
MCV: Mean corpuscular volume
MCH: Mean corpuscular hemoglobin
LSK: Lineage^−^ Sca-1^+^ c-kit^+^
Runx2: Runt-related transcription factor 2
Alpl: Alkaline phosphatase liver/bone/kidney
Ctsk: Cathepsin K
Acp5: Acid phosphatase 5
TRAP: Tartrate-resistant acid phosphatase
Col3a: Collagen type III alpha 1
Fn: Fibronectin
Acta2: Actin alpha 2
