# Supplementary material for: Inhibition of TGFβ improves hematopoietic stem cell niche and ameliorates cancer-related anemia
Source: Stem Cell Res Ther. 2021 Jan 18;12:65. doi: 10.1186/s13287-020-02120-9 (PMC7814632; doi:10.1186/s13287-020-02120-9)
Supplement: Supplementary file 1 — Additional file 1: Fig. S1. General characteristics of LLC mice model. a The tumor volume of tumor-bearing mice since LLC implantation (n=6). b Survival probability of control and LLC-bearing mice over time since LLC implantation (n=20). Data are presented as the means ± SD. Fig. S2. Hematopoiesis was influenced in bone marrow of CRA mice. a Cells number in bone marrow of control and LLC-bearing mice (n=6). b The percentage of myeloid cells in bone marrow of control and LLC-bearing mice (n=6). Data are presented as the means ± SD. *P<0.05, ***P<0.001; assessed by Student’s t test. Fig. S3 LLC-bearing mice exhibited imbalanced bone remodeling. a-c Micro-computed tomography (micro-CT) analysis of trabecular spacing, trabecular pattern factor and trabecular thickness of trabecular bone from the distal femur metaphyses of controls and LLC-bearing mice. Data are presented as the means ± SD of three independent experiments. *P<0.05, **P<0.01; assessed by Student’s t test. Fig. S4 The number of osteoclasts and osteoblasts was increased in cancer cachexia mice. a TRAP stain showing the osteoclasts in trabecular bone from control and LLC-bearing mice. Scale bar, 100 μm. b Immunofluorescence staining of Runx2 showing the osteoblasts in trabecular bone from control and LLC-bearing mice. Scale bar, 100 μm. Fig. S5 Active form of TGFβ1 was increased in CRA mice. a The concentration of active TGFβ1 in the serum of controls and LLC-bearing mice. Data are presented as the means ± SD of three independent experiments. ***P<0.001; assessed by Student’s t test. Fig. S6 SB505124 alleviated the hindered hematopoiesis in LLC-bearing mice. a Experimental design illustrating subcutaneous injection of DMSO or SB505124 (5 mg/day/kg) into control and LLC-bearing mice (n=6/group). b-e The percentage and number of Ter119+ cells in cluster III and cluster V in bone marrow of control, LLC and LLC+SB505124 mice. f The number of white blood cells in peripheral blood of control, LLC and LLC+SB505124 [file 13287_2020_2120_MOESM1_ESM.docx]

**Supplementary materials**

**Inhibition of TGF-β improves hematopoietic stem cell niche and ameliorates cancer-related anemia**

Boyan Wang^1,2,8^, Yi Wang^1,2,8^, Hainan Chen^1,2,8^, Senyu Yao^1,2^, Xiaofan Lai^2,3^, Yuan Qiu^1,2^, Jianye Cai^2,4^, Yinong Huang^2,5^, Xiaoyue Wei^1,2^, Yuanjun Guan^6^, Tao Wang^1,2^, Jiancheng Wang^1,2,*^, Andy Peng Xiang^1,2,7,*^

*Correspondence:

Dr. Andy Peng Xiang, E-mail: xiangp@mail.sysu.edu.cn

Dr. Jiancheng Wang, E-mail: wangjch38@mail.sysu.edu.cn

**Fig. S1**


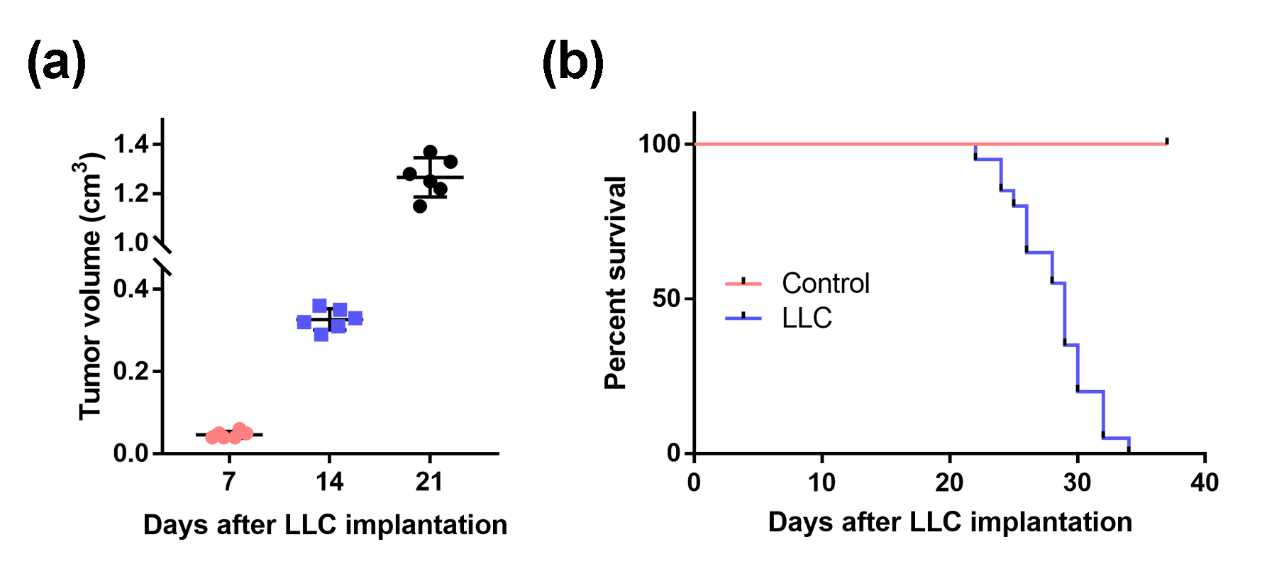


**Fig. S1.** General characteristics of LLC mice model. **a** The tumor volume of tumor-bearing mice since LLC implantation (n=6). **b** Survival probability of control and LLC-bearing mice over time since LLC implantation (n=20). Data are presented as the means ± SD.

**Fig. S2**


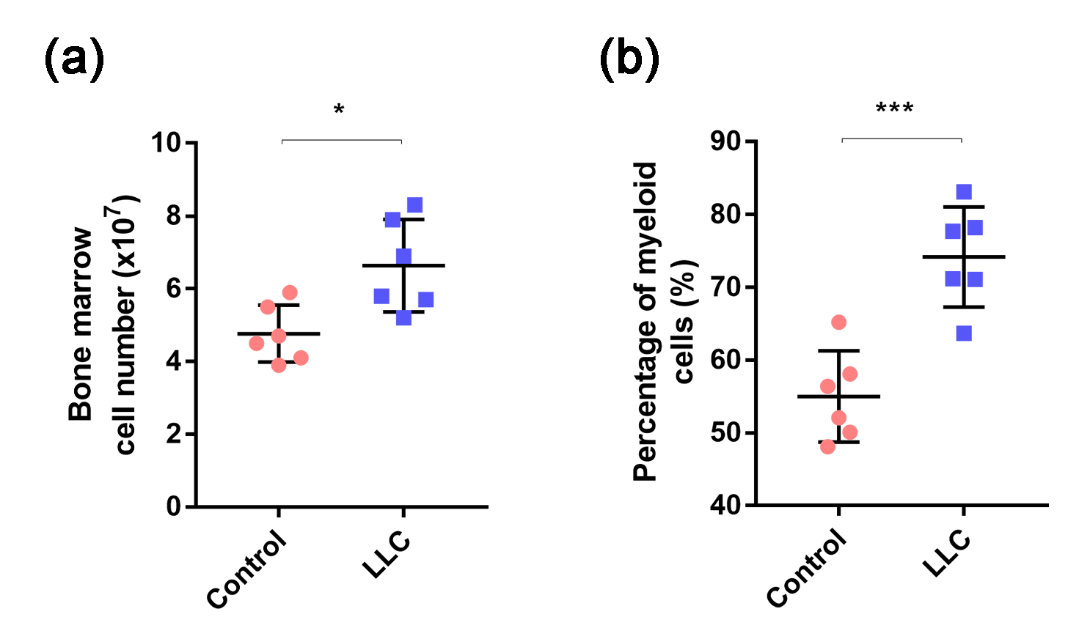


**Fig. S2.** Hematopoiesis was influenced in bone marrow of CRA mice. **a** Cells number in bone marrow of control and LLC-bearing mice (n=6). **b** The percentage of myeloid cells in bone marrow of control and LLC-bearing mice (n=6). Data are presented as the means ± SD. *P<0.05, ****P*<0.001; assessed by Student’s *t* test.

**Fig. S3**


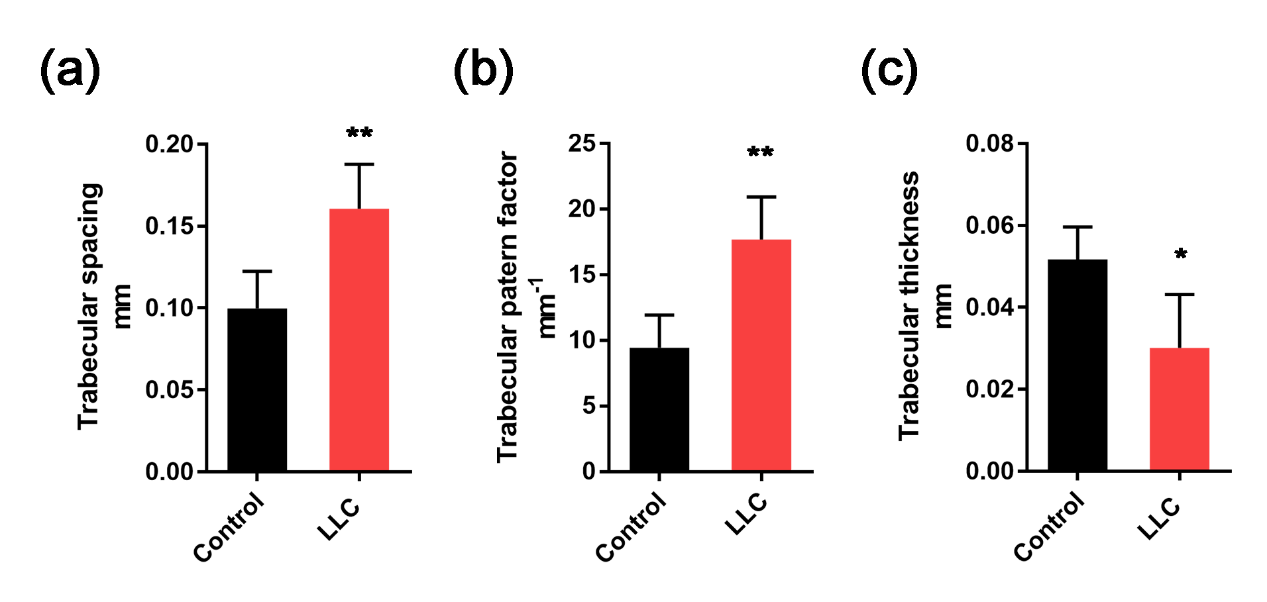


**Fig. S3** LLC-bearing mice exhibited imbalanced bone remodeling. **a-c** Micro-computed tomography (micro-CT) analysis of trabecular spacing, trabecular pattern factor and trabecular thickness of trabecular bone from the distal femur metaphyses of controls and LLC-bearing mice. Data are presented as the means ± SD of three independent experiments. **P*<0.05, ***P*<0.01; assessed by Student’s *t* test.

**Fig. S4**


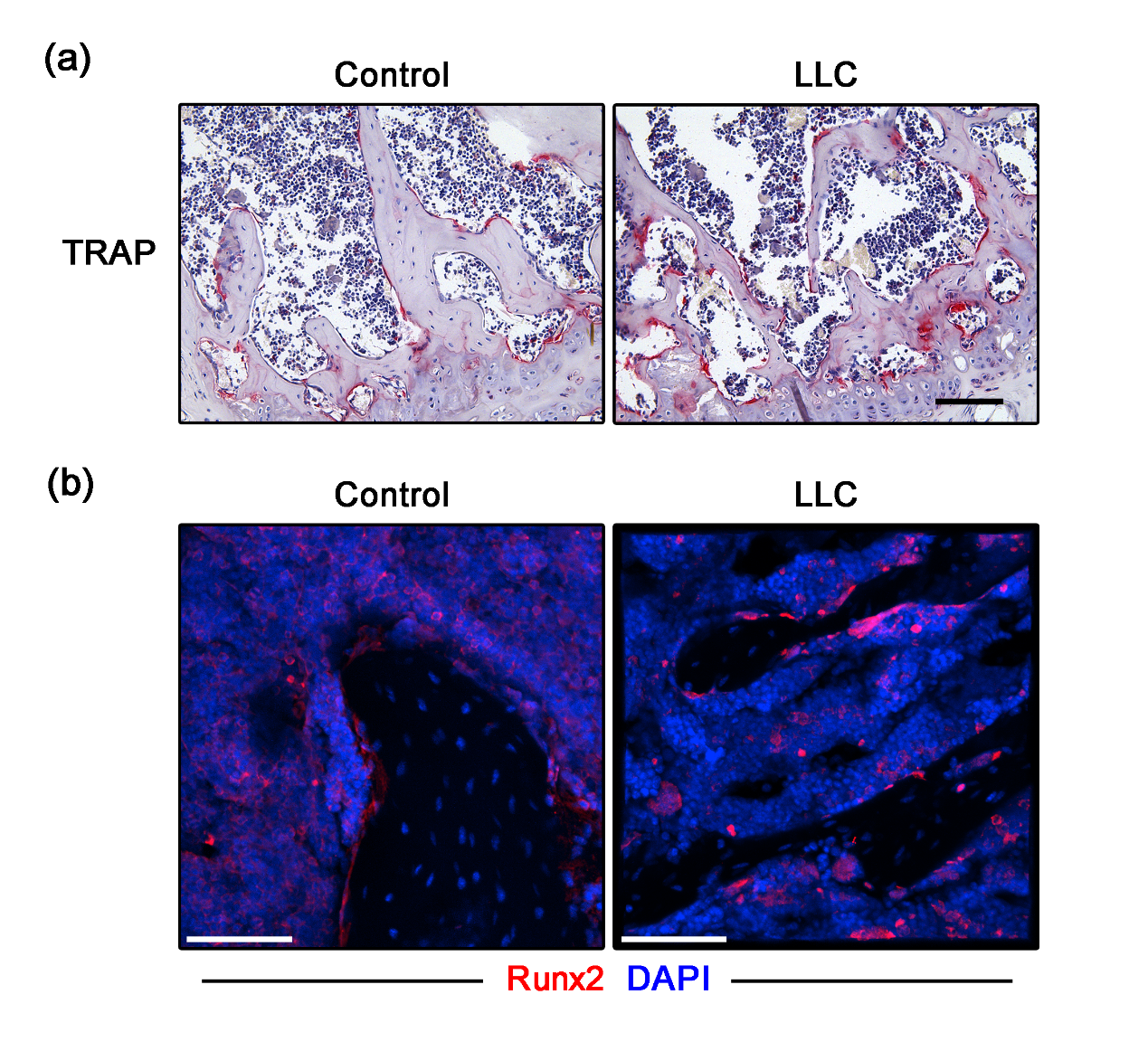


**Fig. S4** The number of osteoclasts and osteoblasts was increased in cancer cachexia mice. **a** TRAP stain showing the osteoclasts in trabecular bone from control and LLC-bearing mice. Scale bar, 100μm. **b** Immunofluorescence staining of Runx2 showing the osteoblasts in trabecular bone from control and LLC-bearing mice. Scale bar, 100μm.

**Fig. S5**


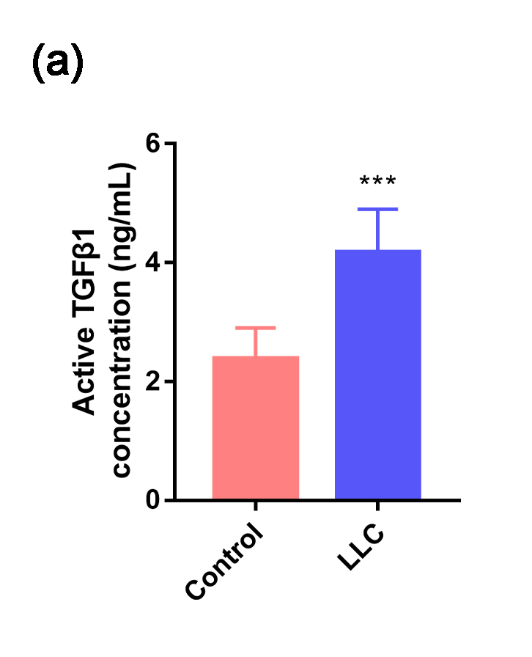


**Fig. S5** Active form of TGFβ1 was increased in CRA mice. **a** The concentration of active TGFβ1 in the serum of controls and LLC-bearing mice. Data are presented as the means ± SD of three independent experiments. ****P*<0.001; assessed by Student’s *t* test.

**Fig. S6**


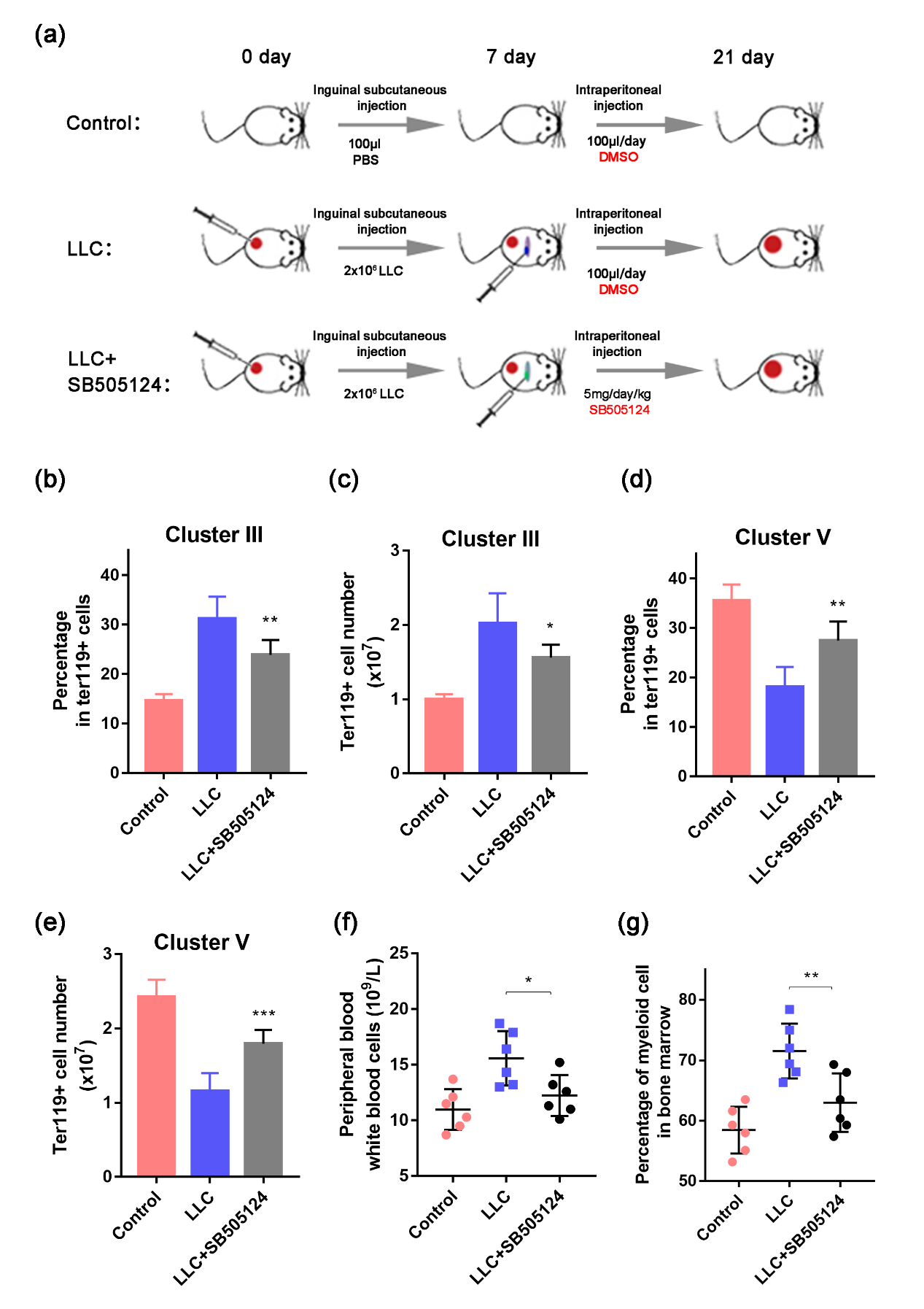


**Fig. S6** SB505124 alleviated the hindered hematopoiesis in LLC-bearing mice. **a** Experimental design illustrating subcutaneous injection of DMSO or SB505124 (5 mg/day/kg) into control and LLC-bearing mice (n=6/group). **b-e** The percentage and number of Ter119+ cells in cluster III and cluster V in bone marrow of control, LLC and LLC+SB505124 mice. **f** The number of white blood cells in peripheral blood of control, LLC and LLC+SB505124 mice. **g** The percentage of myeloid cells in bone marrow of control, LLC and LLC+SB505124 mice. Data are presented as the means ± SD of three independent experiments. **P*<0.05, ***P*<0.01; assessed by Student’s *t* test.

**Fig. S7**


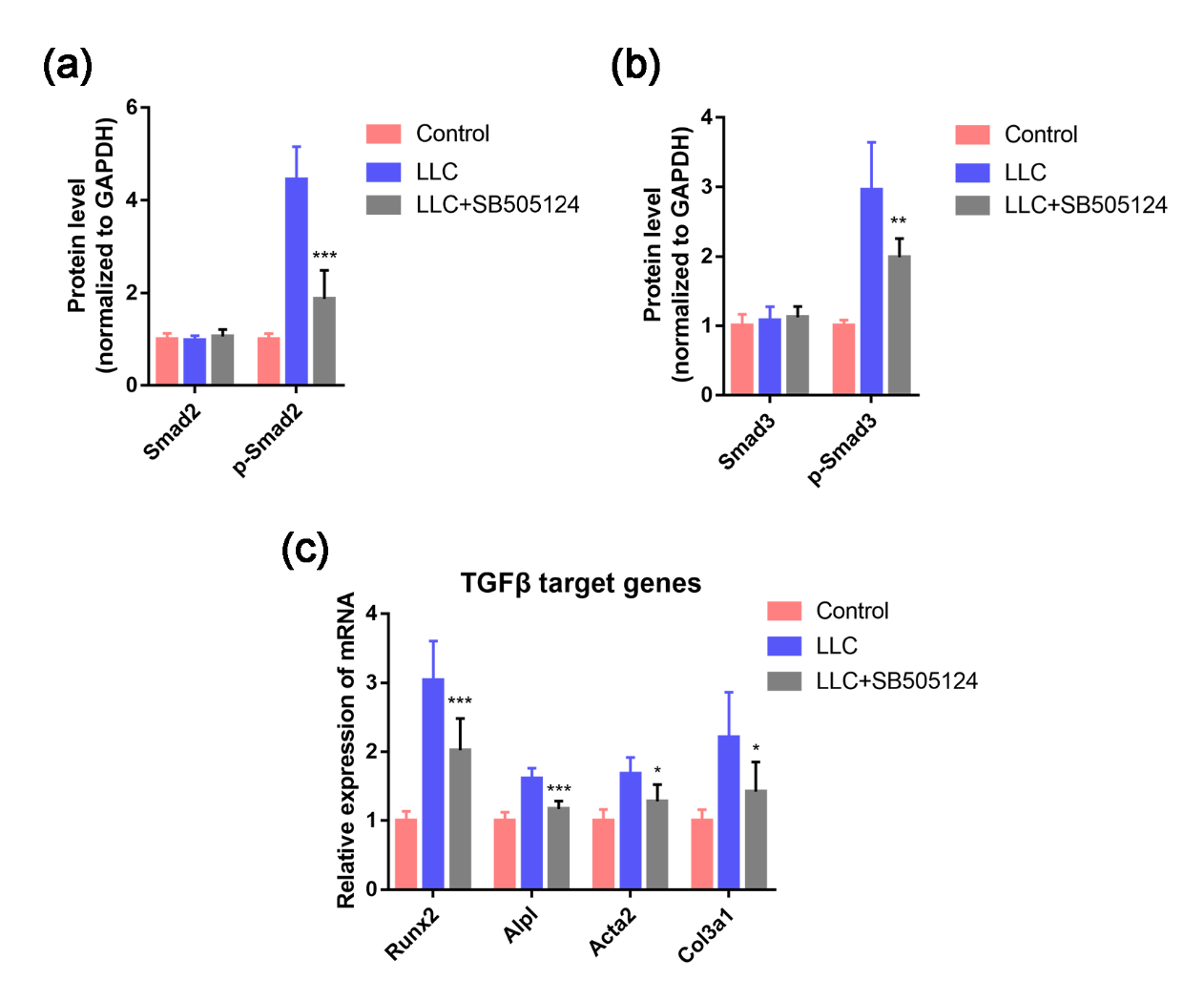


**Fig. S7** SB505124 inhibited TGFβ signaling activation in bone marrow and MSC. **a-b** Quantifications of the Western blotting of phosphorylated Smad2/3 and total Smad2/3 proteins in bone marrow of control, LLC and LLC+SB505124 mice (n=6/group). **c** mRNA expression of TGFβ target genes in MSC of control, LLC and LLC+SB505124 mice (n=6/group). Data are presented as the means ± SD of three independent experiments. **P*<0.05; ***P*<0.01; ****P*<0.001; assessed by Student’s *t* test.

**Fig. S8**


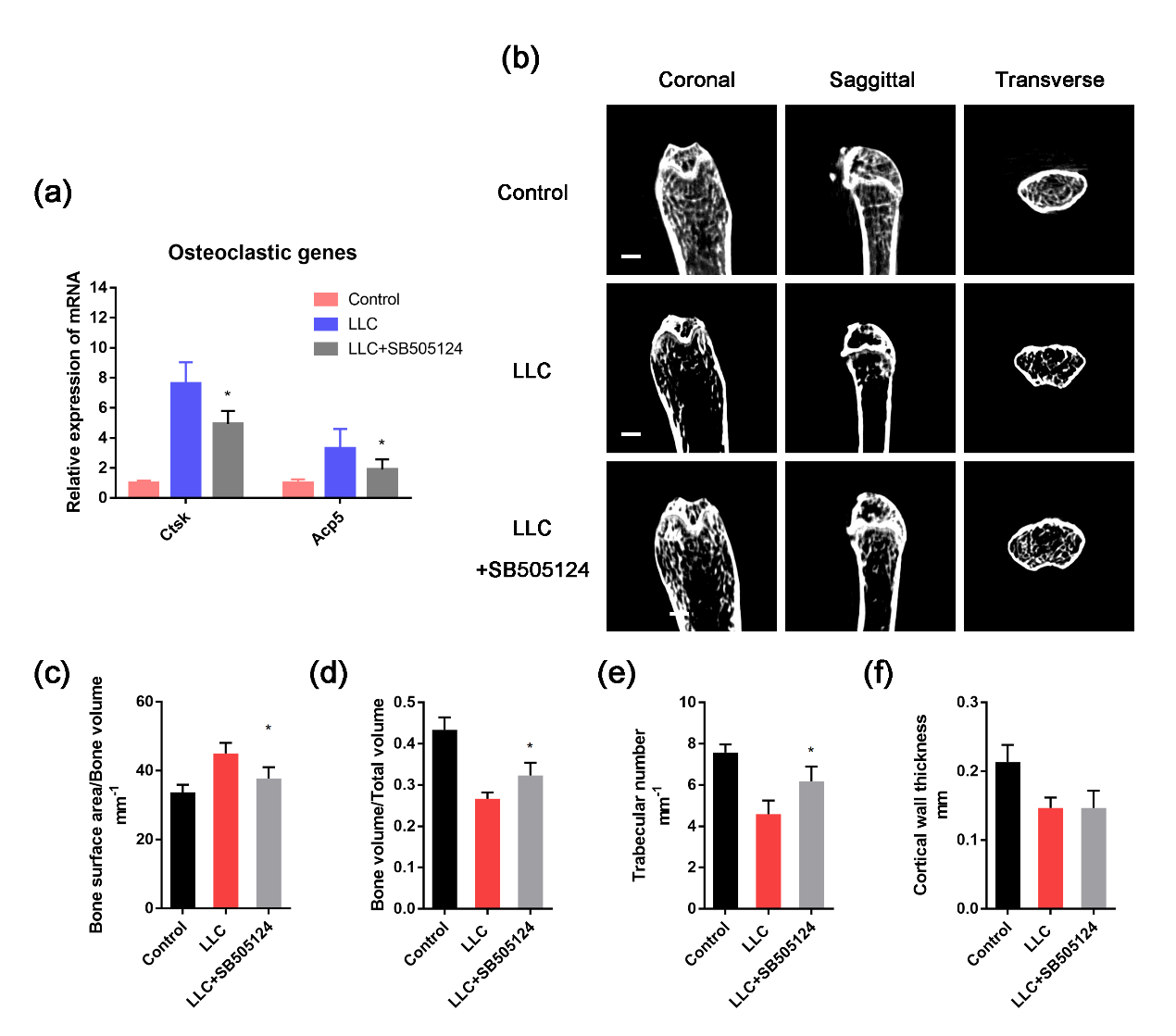


**Fig. S8** SB505124 relieved the osteolytic process of LLC-bearing mice. **a** The mRNA levels of osteoclastic genes in bone marrow of control, LLC and LLC+SB505124 mice (n=6/group). **b** Representative three-dimensional thickness maps from micro-CT scans of trabecular bone from the distal femur metaphysis of control, LLC and LLC+SB505124 mice. Scale bar, 1000 μm. **c-f** Ratio of bone surface area to bone volume, ratio of bone volume to total volume, trabecular number, and cortical wall thickness of control, LLC and LLC+SB505124 mice (n=3/group). Data are presented as the means ± SD of three independent experiments. **P*<0.05; assessed by Student’s *t* test.

**Supplemental Table S1**

**Primer used to amplify the mouse transcripts during PCR.**

| **Gene** | **Sequence (5’-3’)** | **Application** |
| --- | --- | --- |
| ***Kitl*** | Forward: ATGAAGAAGACACAAACTTGGATT  Reverse: CTAGGCAAAACATCCATCCCG | qRT-PCR |
| ***Cxc12*** | Forward: CGGTTCTTCGAGAGCCACAT  Reverse: CCAGGTACTCTTGGATCCACTT | qRT-PCR |
| ***Runx2*** | Forward: GACTGTGGTTACCGTCATGGC  Reverse: ACTTGGTTTTTCATAACAGCGGA | qRT-PCR |
| ***Alpl*** | Forward: CCAACTCTTTTGTGCCAGAGA  Reverse: GGCTACATTGGTGTTGAGCTTTT | qRT-PCR |
| ***Acp5*** | Forward: CGTCTCTGCACAGATTGCAT  Reverse: AAGCGCAAACGGTAGTAAGG | qRT-PCR |
| ***Ctsk*** | Forward: GGAGGCGGCTATATGACCA  Reverse: GGCGTTATACATACAACTTTCATCC | qRT-PCR |
| ***Acta2*** | Forward: CTGACAGAGGCACCACTGAA  Reverse: CATCTCCAGAGTCCAGCACA | qRT-PCR |
| ***Col3a1*** | Forward: ACGTAGATGAATTGGGATGCAG  Reverse: GGGTTGGGGCAGTCTAGTG | qRT-PCR |
| ***Ppar-γ*** | Forward: CTGACCCAATGGTTGCT  Reverse: CAGACTCGGCACTCAATG | qRT-PCR |
| ***Fabp4*** | Forward: AATCACCGCAGACGACA  Reverse: GTGGAAGTCACGCCTTTC | qRT-PCR |
| ***Fn*** | Forward: ATGTGGACCCCTCCTGATAGT  Reverse: GCCCAGTGATTTCAGCAAAGG | qRT-PCR |
| ***18s*** | Forward: GTAACCCGTTGAACCCCATT  Reverse: CCATCCAATCGGTAGTAGCG | qRT-PCR |

**Supplemental Table S2**

**Primary and secondary antibodies**

| **Name** | **Company** | **Dilution** | **Cat.** |
| --- | --- | --- | --- |
| CD44 Monoclonal Antibody (IM7), APC | eBioscience | 1:100 | 17-0441-82 |
| TER-119 Monoclonal Antibody (TER-119), PE-Cyanine7 | eBioscience | 1:100 | 25-5921-82 |
| Mouse Hematopoietic Lineage Antibody Cocktail, eFluor 450 | eBioscience | 1:100 | 88-7772-72 |
| Ly-6A/E (Sca-1) Monoclonal Antibody (D7), APC | eBioscience | 1:100 | 17-5981-82 |
| CD117 (c-Kit) Monoclonal Antibody (2B8), APCeFluor 780 | eBioscience | 1:100 | 47-1171-82 |
| CD16/CD32 Monoclonal Antibody (93) | eBioscience | 1:100 | 14-0161-81 |
| CD127 Monoclonal Antibody (A7R34), PE-Cyanine7 | eBioscience | 1:100 | 25-1271-82 |
| CD135 (Flt3) Monoclonal Antibody (A2F10), PE | eBioscience | 1:100 | 12-1351-82 |
| CD16/CD32 Monoclonal Antibody (93), Alexa Fluor 700 | eBioscience | 1:100 | 56-0161-82 |
| CD34 Monoclonal Antibody (RAM34), FITC | eBioscience | 1:100 | 11-0341-82 |
| CD31 (PECAM-1) Monoclonal Antibody (390), PE-Cyanine7 | eBioscience | 1:100 | 25-0311-82 |
| CD45 Monoclonal Antibody (30-F11), PE-Cyanine5 | eBioscience | 1:100 | 15-0451-82 |
| Runx2 | Santa Cruz | 1:100 | sc-390351 |
| Anti-Smooth Muscle Actin Antibody | abcam | 1:200 | ab5694 |
| Anti-Phospho-Smad 2/3 Antibody | Santa Cruz | 1:100 | sc-11769 |
| Anti-Phospho-Smad2 Antibody | CST | 1:1000 | 3108 |
| Anti-Phospho-Smad3 Antibody | CST | 1:1000 | 9520 |
| Anti-Smad2 Antibody | CST | 1:1000 | 5339 |
| Anti-Smad3 Antibody | CST | 1:1000 | 9523 |
| Anti-Rabbit IgG HRP-linked Ab | CST | 1:5000 | 7074 |
| Goat Anti-Rabbit IgG Alexa 555 | Invitrogen | 1:500 | A21428 |
| Donkey Anti-Goat IgG Alexa 594 | Invitrogen | 1:500 | A11058 |
